# Admission interleukin-6 as a time-limited, etiology-dependent prognostic signal in hospitalized cirrhosis – A frequentist and Bayesian cohort study

**DOI:** 10.1371/journal.pone.0360005

**Published:** 2026-10-08

**Authors:** Tharun Tom Oommen, Cyriac Abby Philips, Arif Hussain Theruvath, Resmi Ravindran, Aryalakshmi Sreemohan, Ambily Baby, Rizwan Ahamed, Jaicob Varghese, Biji Thomas Philip, Ajit Tharakan, Philip Augustine

**Affiliations:** 1 Department of Clinical and Translational Hepatology, The Liver Institute, Center of Excellence in Gastrointestinal Sciences, Rajagiri Hospital, Kochi, Kerala, India; 2 Department of Gastroenterology and Advanced GI Endoscopy, Center of Excellence in Gastrointestinal Sciences, Rajagiri Hospital, Kochi, Kerala, India; 3 Clinical Research Division, The Liver Institute, Center of Excellence in Gastrointestinal Sciences, Rajagiri Hospital, Kochi, Kerala, India; 4 Department of Critical Care Medicine, Rajagiri Hospital, Kochi, Kerala, India; Charite Universitatsmedizin Berlin, GERMANY

## Abstract

**Background and objectives:**

Interleukin-6 is a proximal inflammatory cytokine implicated in cirrhosis decompensation, yet its incremental prognostic value over established severity scores remains unclear.

**Methods:**

In this prospective, purely observational study (April 2022 to October 2023), 489 consecutive patients hospitalized with cirrhosis at a tertiary hepatology center were enrolled, and serum interleukin-6 was measured at admission.

**Results:**

Median interleukin-6 was 23.8 pg/mL, in-hospital mortality was 9.0%, and overall mortality was 70.6%. Interleukin-6 independently predicted in-hospital mortality after adjustment for age, sex, and disease severity (adjusted odds ratio 1.34, 95% confidence interval 1.08–1.67), supported by Bayesian analysis (posterior probability 99.6%). Adding interleukin-6 to conventional severity scores modestly but significantly improved discrimination for in-hospital mortality, with the combined organ failure score plus interleukin-6 model achieving the highest area under the curve (0.802). The prognostic impact of interleukin-6 was substantially stronger in alcohol-associated liver disease compared with metabolic dysfunction-associated steatotic liver disease. Infection mediated 17.3% of the interleukin-6–mortality association. In sub-cohort analyses, the etiology-specific effect persisted after adjustment for infection and after exclusion of severe alcohol-associated hepatitis; infection mediated 23% of the interleukin-6–mortality association in alcohol-associated liver disease but 2% in metabolic dysfunction-associated steatotic liver disease, and the incremental value of interleukin-6 over MELD-3.0 was confined to alcohol-associated disease and to infected patients. MELD-3.0 remained the dominant prognostic determinant, and interleukin-6's independent prognostic signal attenuated for longer-term outcomes.

**Conclusions:**

Admission interleukin-6 did not outperform established severity scores but provided modest, statistically significant incremental discrimination that was confined to the in-hospital window and concentrated in alcohol-associated disease. These findings do not support routine interleukin-6 measurement in unselected admissions but identify alcohol-associated cirrhosis, particularly with infection or intermediate MELD-3.0 scores, as the setting in which interleukin-6-informed risk stratification merits prospective validation and economic evaluation.

## 1. Introduction

Cirrhosis represents a major global health burden, accounting for approximately two million deaths annually and responsible for 4% of all deaths worldwide. The Global Burden of Disease Study estimates 10.6 million prevalent cases of decompensated cirrhosis and 112 million cases of compensated cirrhosis globally, with mortality rates 5- to 10-fold higher than the general population [1,2]. Despite advances in disease-modifying therapies, hospitalized patients with cirrhosis continue to experience high short-term mortality, particularly those who develop acute decompensation or acute-on-chronic liver failure (ACLF). Acute-on-chronic liver failure is a distinct clinical syndrome characterized by acute deterioration of liver function in patients with chronic liver disease, frequently complicated by extrahepatic organ failures and high short-term mortality reaching 50–90% in severe cases [3]. The pathophysiology of ACLF is increasingly recognized to involve a dysregulated systemic inflammatory response, wherein circulating levels of proinflammatory cytokines and chemokines are markedly elevated and correlate with the extent of organ dysfunction and mortality [4,5]. Among hospitalized patients with cirrhosis, the presence and severity of systemic inflammation – rather than liver dysfunction alone, may be a critical determinant of clinical outcomes.

Interleukin-6 (IL-6) is a pleiotropic cytokine that occupies a central position in the inflammatory cascade and acute-phase response. Produced primarily by macrophages, hepatocytes, and Kupffer cells in response to infection and tissue injury, IL-6 stimulates hepatic synthesis of acute-phase reactants including C-reactive protein (CRP), complement components, and coagulation factors [6,7]. In cirrhosis, elevated IL-6 reflects both hepatic inflammation and extrahepatic sources such as bacterial translocation from the gut, and its levels increase progressively with disease severity from compensated cirrhosis to decompensated states and ACLF [8,9]. Unlike CRP and procalcitonin, which are downstream effectors of the inflammatory response, IL-6 is a proximal mediator that may provide earlier and more direct information about the inflammatory milieu driving clinical deterioration [10].

Several studies have examined IL-6 as a prognostic biomarker in cirrhosis. Remmler and colleagues demonstrated that elevated IL-6 independently predicted 90-day and 1-year mortality in patients with end-stage liver disease awaiting transplantation, with discriminative performance comparable to the Model for End-Stage Liver Disease (MELD) score [11]. Subsequent investigations have confirmed associations between IL-6 and mortality in acute decompensation and HBV-related ACLF [12–14]. A recent Japanese study specifically identified IL-6 as a prognostic factor in alcohol-related ACLF, suggesting potential etiology-specific effects [15].

However, several critical knowledge gaps remain. First, the incremental prognostic value of IL-6 over established scoring systems in real-world hospitalized populations has not been systematically quantified. Second, whether IL-6's prognostic significance varies by etiology – particularly between MASLD and ALD, has not been explored, despite evidence that inflammatory phenotypes differ across etiologies. Third, the mechanistic pathways by which IL-6 influences outcomes – whether primarily through infection, general inflammation, or direct hepatotoxicity – remain unclear. Fourth, optimal clinical cutoff thresholds for risk stratification and the subgroups in whom IL-6 measurement adds most value to routine prognostic assessment have not been defined. Recent efforts to improve conventional scores have focused on adding inflammation-related variables. In a meta-analysis of 32 cohorts comprising 13,939 patients with ACLF, followed by validation in 751 prospectively enrolled patients, Yu et al. showed that adding age, neutrophil count and hepatic encephalopathy grade to MELD (the CATCH-LIFE-MELD score) improved short-term prognostication over MELD, MELD-Na and CLIF-C ACLF scores, in derivation and in an external cohort [16]. That work was conducted in predominantly hepatitis B–related ACLF and used a cellular marker of inflammation; whether an upstream cytokine such as IL-6 adds prognostic information in hospitalized patients with non-viral cirrhosis, and in which subgroups, has not been established.

Given these gaps, we conducted a comprehensive analysis of IL-6 at admission in a large cohort of hospitalized patients with cirrhosis. Specifically, we aimed to: (1) characterize the distribution of IL-6 across clinical phenotypes and disease severity strata; (2) evaluate the independent prognostic value of IL-6 for multiple mortality endpoints after adjustment for established prognostic scores; (3) quantify the incremental discriminative improvement afforded by adding IL-6 to existing risk models; (4) identify clinical subgroups in which IL-6 provides the greatest added prognostic value; (5) explore potential mediating pathways (infection, systemic inflammation) underlying the IL-6–mortality relationship; and (6) determine optimal IL-6 cutoffs for clinical risk stratification.

## 2. Patients

### 2.1. Setting and patient pathways

The study enrolled consecutive patients (April 2022 to October 2023) with cirrhosis hospitalized at a tertiary hepatology center. Patient enrolment was prospective, and the consecutive design was adopted to minimize selection bias. The study is reported in accordance with the Strengthening the Reporting of Observational Studies in Epidemiology (STROBE) statement for cohort studies. This prospective observational study did not involve the assignment of any intervention by the investigators. Inclusion criteria were: (1) age ≥ 18 years; (2) a diagnosis of cirrhosis as defined in Section 2.2; (3) hospitalisation at the study centre between April 2022 and October 2023; and (4) measurement of serum IL-6 within 24 hours of admission as part of the standard admission laboratory panel. Exclusion criteria were (1) absence of liver disease and non-cirrhotic portal hypertension; (2) prior liver transplantation; (3) IL-6 not measured as per protocol; (4) patients admitted for elective procedures and evaluations were eligible provided they met the criteria. This study was conducted in accordance with the ethical principles of the Declaration of Helsinki and its pertinent revisions and was approved by the Institutional Ethics Committee of Rajagiri Hospital, Aluva, India (Approval No.: RAJH/2022/36; Date: 09 March 2022). The Ethics Committee approved a waiver of written informed consent on the basis that: (i) the study was purely observational and did not involve any deviation from routine clinical care; (ii) serum IL-6 was measured from blood samples collected as part of standard clinical evaluation at admission per Department protocols; (iii) no additional interventions, procedures, or blood draws were performed for research purposes; and (iv) all data were anonymized prior to analysis. The study protocol was reviewed and approved prior to enrolment of the first participant.

The primary outcome was in-hospital all-cause mortality. Secondary outcomes included mortality at 6 months, 12 months, and overall mortality during the entire follow-up period. Time-to-event was calculated from the date of hospital admission to the date of death from any cause or last known follow-up, whichever occurred first. Patients alive at the end of the observation period were censored at the date of last contact. In-hospital mortality was analysed as a binary outcome; the time-to-event outcomes were analysed using both binary (logistic) and survival (Cox) approaches.

### 2.2. Definitions

Cirrhosis was diagnosed based on a combination of clinical, biochemical, and imaging criteria, and/or histology when available. Compensated cirrhosis was defined as cirrhosis without current or prior episodes of ascites, variceal haemorrhage, hepatic encephalopathy (HE), or jaundice attributable to liver dysfunction. Decompensated cirrhosis was defined as cirrhosis with one or more of these complications, in accordance with the Baveno VII consensus. Acute-on-chronic liver failure (ACLF) was diagnosed and graded (grades 1–3) using the European Association for the Study of the Liver–Chronic Liver Failure (EASL-CLIF) Consortium criteria, based on the presence and number of organ failures as assessed by the CLIF-C Organ Failure (CLIF-C OF) score. Disease severity was quantified using the Child–Turcotte–Pugh (CTP) classification, the Model for End-Stage Liver Disease 3.0 (MELD-3.0) incorporating sodium and sex-based coefficients, and the Chronic Liver Failure-Consortium Acute Decompensation (CLIF-C AD) score. HE was graded according to the West Haven criteria. Acute kidney injury (AKI) was defined per the International Club of Ascites (ICA) revised criteria as an increase in serum creatinine ≥0.3 mg/dL within 48 hours or ≥50% from a stable baseline within the preceding three months. Infection at admission was defined as a clinically, microbiologically and/or radiologically documented infection present at, or diagnosed within 48 hours of, admission, using the site-specific criteria – Bloodstream infection: growth of a recognised pathogen in at least one blood culture, or of a common skin commensal in at least two separately drawn cultures, with compatible clinical features; Urinary tract infection: urinary or systemic symptoms with >10 leukocytes per high-power field on urinalysis and a positive urine culture (≥10^5^ colony-forming units/mL), or, in catheterised patients, a positive culture with pyuria; pneumonia and lower respiratory tract infection: a new or progressive pulmonary infiltrate on chest imaging plus at least two of fever or hypothermia, leukocytosis or leukopenia, purulent sputum and worsening oxygenation; spontaneous bacterial peritonitis: an ascitic-fluid polymorphonuclear count ≥250 cells/mm³ in the absence of a surgically treatable intra-abdominal source, irrespective of the culture result; and skin and soft-tissue infection: clinical cellulitis, erysipelas or necrotising infection (localised erythema, warmth, tenderness and swelling with or without fever), with or without a positive wound or tissue culture. Blood, urine and ascitic-fluid cultures and chest imaging were obtained before antibiotics in all patients with suspected infection. Empirical therapy followed the departmental protocol, aligned with the standard regional guidance, adapted to local antimicrobial resistance patterns: For community-acquired spontaneous bacterial peritonitis, urinary tract infection and pneumonia, a third-generation cephalosporin (ceftriaxone), with a macrolide added for pneumonia; for healthcare-associated or nosocomial infections and for septic shock, piperacillin–tazobactam or a carbapenem (meropenem), with a glycopeptide (vancomycin or teicoplanin) when methicillin-resistant Staphylococcus aureus or enterococcal infection was suspected, and an echinocandin in septic shock with risk factors for invasive candidiasis; de-escalation to pathogen-directed therapy within 48–72 hours according to culture results; for spontaneous bacterial peritonitis, intravenous albumin 1.5 g/kg on day 1 and 1 g/kg on day 3 in addition to antibiotics, followed by secondary prophylaxis with norfloxacin; for skin and soft-tissue infection, a cephalosporin or amoxicillin–clavulanate, with clindamycin or vancomycin for suspected MRSA and urgent surgical debridement for necrotising infection; for bloodstream infection, pathogen-directed therapy for 10–14 days with removal of intravascular devices; for urinary tract infection, a third-generation cephalosporin or a fluoroquinolone for 5–7 days according to susceptibility; for pneumonia, 7 days of therapy with review of oxygen requirement and ventilatory support.

Alcohol-associated liver disease (ALD) was defined as liver disease attributable to significant alcohol consumption (>40 g/day for men, > 20 g/day for women for ≥5 years). Severe alcohol-associated hepatitis (sAH) was defined according to the National Institute on Alcohol Abuse and Alcoholism (NIAAA) Alcoholic Hepatitis Consortia criteria and the 2019 AASLD practice guidance. Severe AH was diagnosed in patients with a Maddrey discriminant function ≥32. Metabolic dysfunction-associated steatotic liver disease (MASLD) was defined according to the 2023 multi-society Delphi consensus nomenclature as hepatic steatosis in the presence of at least one cardiometabolic risk factor.

## 3. Statistics

### 3.1. Basic statistics

Continuous variables were summarized as median with interquartile range, and categorical variables as counts and percentages. Natural logarithm transformation was applied for use in parametric models, and patients were additionally categorized into tertiles and quartiles for stratified analyses. Between-group comparisons were performed using the Mann–Whitney U test and Kruskal–Wallis test for continuous variables, and the chi-squared or Fisher's exact test for categorical variables. Bivariate associations between IL-6 and clinical parameters were assessed using Spearman rank correlation coefficients. Receiver operating characteristic curves were constructed for each mortality endpoint, with AUCs estimated by the DeLong method; optimal cutoffs were determined by Youden's index and at prespecified specificity thresholds. No formal a priori sample-size calculation was performed; the sample comprised all consecutive eligible admissions during the study period.

### 3.2. Advanced statistics

Univariate and multivariable binary logistic regression models estimated the association between log-transformed IL-6 and in-hospital, 6-month, 12-month, and overall mortality, with the primary adjusted model controlling for age, sex, and disease severity; model calibration was assessed by the Hosmer–Lemeshow test, and internal validation was performed using 500-iteration bootstrap resampling to derive an optimism-corrected C-statistic. Kaplan–Meier survival analysis with log-rank testing and Cox proportional hazards regression (with verification of the proportional hazards assumption via Schoenfeld residuals) were used for time-to-event outcomes, supplemented by landmark analyses at 30 and 90 days. Incremental predictive value of IL-6 over disease severity scoring system was quantified using the likelihood ratio test, ΔAUC (area under the curve analysis), and integrated discrimination improvement. Subgroup-specific discrimination and formal multiplicative interaction testing were performed across predefined clinical strata. Mediation analyses were performed using the Baron and Kenny framework. A classification and regression tree (CART) model explored non-linear hierarchical predictor relationships. The tree was used for exploratory purposes to identify clinically interpretable decision rules and to assess the relative importance of IL-6 compared with established prognostic scores in a non-parametric framework. Bayesian logistic regression with weakly informative priors [Normal (0,1)] and Hamiltonian Monte Carlo sampling provided posterior odds ratios with 95% credible intervals, posterior probabilities of association, and model comparison via Widely Applicable Information Criterion (WAIC) and leave-one-out cross-validation using Pareto-smoothed importance sampling (LOO-CV). Sensitivity analyses excluding extreme variable values (≥99th percentile) were performed to confirm the robustness of all primary findings.

Post hoc sub-cohort analyses requested at peer review examined IL-6 separately in patients with documented infection at admission, in patients with sAH, in the combination of the two, and within etiology strata, using the same logistic-regression and ROC methods; the Baron and Kenny mediation analysis and the IL-6 × etiology interaction were repeated within strata and after adjustment for infection and MELD-3.0. Where a stratum contained fewer than 10 outcome events, Firth's bias-reduced penalised-likelihood logistic regression with profile-likelihood confidence intervals was used, and MELD-adjusted estimates were not attempted with fewer than five events. These analyses were exploratory, were not corrected for multiplicity, and are interpreted as hypothesis-generating.

All statistical analyses were performed using Python (version 3.10; Python Software Foundation, Wilmington, DE, USA). Data management and processing were conducted using pandas (version 2.1) and NumPy (version 1.26). Descriptive and inferential statistics, including the Mann–Whitney U test, Kruskal–Wallis test, chi-squared test, and Spearman rank correlation, were computed using SciPy (version 1.11). Multivariable logistic regression, variance inflation factor assessment, and calibration analyses were performed using statsmodels (version 0.14). Receiver operating characteristic curve analysis, including area under the curve computation and Youden index–based optimal cutoff determination, was conducted using scikit-learn (version 1.3). Kaplan–Meier survival estimation, log-rank testing, and Cox proportional hazards regression were performed using the lifelines package (version 0.27). Bayesian logistic regression models were fitted using PyMC (version 5.10) with Markov chain Monte Carlo sampling under weakly informative priors. Net reclassification improvement and integrated discrimination improvement were calculated using custom implementations. All figures were generated using Matplotlib (version 3.8) and Seaborn (version 0.13). A two-sided p-value < 0.05 was considered statistically significant for frequentist analyses; for Bayesian analyses, posterior probabilities ≥ 95% were considered strong evidence of association.

## 4. Results

### 4.1. Study population and baseline characteristics

During the study period, 828 consecutive admissions of patients with suspected or known liver disease were screened; 339 were excluded (age < 18 years, N = 23; no liver disease, N = 66; non-cirrhotic liver disease, N = 183; IL-6 not measured at admission, N = 56; prior liver transplantation, N = 11), leaving 489 patients for analysis. The cohort was predominantly male (418/489, 85.5%) with a median age of 62 years [interquartile range (IQR) 54–68]. The most common clinical syndrome was decompensated cirrhosis (338/489, 69.1%), followed by ACLF (112/489, 22.9%) and compensated cirrhosis (39/489, 8.0%). Metabolic dysfunction-associated steatotic liver disease (MASLD) was the predominant etiology (306/489, 62.6%), followed by alcohol-associated liver disease (ALD; 160/489, 32.7%). Comorbid conditions were prevalent: diabetes mellitus in 298 (60.9%), hypertension in 88 (18%), and obesity in 154 (31.5%).

At admission, infection was documented in 145 patients (29.7%), ascites in 267 (54.6%), hepatic encephalopathy (HE) in 131 (26.8%) and acute kidney injury (AKI) in 103 (21.1%). By CLIF-C OF–based organ-failure grading (Table 1), 104 patients (21.3%) had no organ failure, 124 (25.4%) had grade 1, 185 (37.8%) grade 2, and 76 (15.5%) grade 3. A total of 150 patients (30.7%) required medical intensive care unit (MICU) admission at presentation.

**Table 1:** Baseline Characteristics of Hospitalized Liver Disease Patients by IL-6 Tertiles (N = 489).

Variable	T1 (Low)n = 163	T2 (Medium)n = 163	T3 (High)n = 163	OverallN = 489	p-value
**Demographics**					
Age (years)	60.0 (54.5–67.0)	62.0 (53.0–68.0)	63.0 (53.5–68.5)	62.0 (54.0–68.0)	0.559
Male sex	137 (84.0%)	143 (87.7%)	138 (84.7%)	418 (85.5%)	0.600
**IL-6 (pg/mL)**					
Median (IQR)	5.3 (2.8–9.4)	23.8 (17.6–33.4)	121.0 (71.9–244.9)	23.8 (9.4–71.8)	**<0.001**
**Clinical Syndrome**					
Compensated cirrhosis	22 (13.5%)	10 (6.1%)	7 (4.3%)	39 (8.0%)	0.005
Decompensated cirrhosis	114 (69.9%)	113 (69.3%)	111 (68.1%)	338 (69.1%)	0.935
Acute-on-chronic liver failure	27 (16.6%)	40 (24.5%)	45 (27.6%)	112 (22.9%)	0.050
**Etiology**					
MASLD	111 (68.1%)	100 (61.3%)	95 (58.3%)	306 (62.6%)	0.173
ALD	42 (25.8%)	55 (33.7%)	63 (38.7%)	160 (32.7%)	0.044
**ACLF Grade**					
No ACLF	53 (32.5%)	27 (16.6%)	24 (14.7%)	104 (21.3%)	**<0.001**
ACLF 1	57 (35.0%)	44 (27.0%)	23 (14.1%)	124 (25.4%)	**<0.001**
ACLF 2	47 (28.8%)	70 (42.9%)	68 (41.7%)	185 (37.8%)	0.014
ACLF 3	6 (3.7%)	22 (13.5%)	48 (29.4%)	76 (15.5%)	**<0.001**
**Comorbidities**					
Diabetes	109 (66.9%)	100 (61.3%)	89 (54.6%)	298 (60.9%)	0.075
Hypertension	26 (16.0%)	25 (15.3%)	37 (22.7%)	88 (18.0%)	0.158
Obesity	71 (43.6%)	48 (29.4%)	35 (21.5%)	154 (31.5%)	**<0.001**
**Clinical Features**					
MICU admission	39 (23.9%)	39 (23.9%)	72 (44.2%)	150 (30.7%)	**<0.001**
AVB at admission	40 (24.5%)	21 (12.9%)	30 (18.4%)	91 (18.6%)	0.026
HE at admission	28 (17.2%)	41 (25.2%)	62 (38.0%)	131 (26.8%)	**<0.001**
AKI at admission	17 (10.4%)	34 (20.9%)	52 (31.9%)	103 (21.1%)	**<0.001**
Hypotension at admission	4 (2.5%)	4 (2.5%)	23 (14.1%)	31 (6.3%)	**<0.001**
Infection present	23 (14.1%)	40 (24.5%)	82 (50.3%)	145 (29.7%)	**<0.001**
Ascites at admission	62 (38.0%)	106 (65.0%)	99 (60.7%)	267 (54.6%)	**<0.001**
Refractory ascites	21 (12.9%)	49 (30.1%)	62 (38.0%)	132 (27.0%)	**<0.001**
Hyponatremia at admission	43 (26.4%)	69 (42.3%)	66 (40.5%)	178 (36.4%)	0.005
HCC diagnosed	34 (20.9%)	29 (17.8%)	34 (20.9%)	97 (19.8%)	0.725
Number of organ failures (admission)	1.0 (0.0–2.0)	2.0 (1.0–2.0)	2.0 (1.0–3.0)	2.0 (1.0–2.0)	**<0.001**
**Laboratory Values**					
Hemoglobin (g/dL)	10.8 (9.2–12.4)	10.3 (8.6–11.7)	9.8 (8.1–11.5)	10.2 (8.6–11.8)	0.001
TLC (×10³/μL)	5.6 (4.2–8.0)	6.8 (4.4–9.7)	9.4 (6.8–13.3)	7.0 (4.7–10.5)	**<0.001**
Platelet count (×10³/μL)	124.0 (100.0–148.0)	125.0 (90.0–150.0)	130.0 (97.5–140.0)	125.0 (100.0–145.0)	0.745
Total bilirubin (mg/dL)	2.2 (1.2–4.7)	3.6 (1.8–7.9)	4.1 (2.1–9.4)	3.3 (1.6–6.6)	**<0.001**
Direct bilirubin (mg/dL)	0.9 (0.5–2.2)	2.0 (0.9–4.3)	2.2 (1.1–5.1)	1.7 (0.7–3.6)	**<0.001**
AST (IU/L)	59.5 (42.0–87.0)	67.0 (47.0–127.5)	73.0 (48.5–125.5)	66.0 (45.0–115.0)	0.008
ALT (IU/L)	36.0 (26.2–54.8)	41.5 (26.0–65.0)	40.0 (26.5–63.5)	39.0 (26.0–62.0)	0.684
Albumin (g/dL)	3.0 (2.6–3.5)	2.7 (2.3–3.0)	2.6 (2.2–3.0)	2.7 (2.4–3.2)	**<0.001**
INR	1.5 (1.3–1.8)	1.8 (1.5–2.3)	2.0 (1.6–2.5)	1.7 (1.4–2.2)	**<0.001**
Blood urea (mg/dL)	29.9 (19.2–49.0)	34.7 (23.5–55.4)	46.9 (31.5–79.8)	37.4 (23.5–61.3)	**<0.001**
Creatinine (mg/dL)	1.0 (0.8–1.3)	1.1 (0.8–1.6)	1.3 (0.9–2.3)	1.1 (0.8–1.7)	**<0.001**
Sodium (mEq/L)	135.5 (132.0–138.0)	132.0 (128.0–136.0)	131.0 (126.0–136.0)	133.0 (128.0–137.0)	**<0.001**
Potassium (mEq/L)	4.0 (3.7–4.3)	4.0 (3.6–4.4)	4.2 (3.7–4.7)	4.1 (3.6–4.5)	0.005
CRP (mg/L)	6.0 (4.0–13.3)	19.4 (8.2–41.2)	44.1 (19.9–70.5)	18.1 (5.8–44.7)	**<0.001**
Procalcitonin (ng/mL)	0.1 (0.1–0.2)	0.3 (0.1–0.6)	0.9 (0.3–2.6)	0.3 (0.1–0.9)	**<0.001**
**Prognostic Scores**					
CTP score	8.0 (6.0–10.0)	10.0 (8.0–11.0)	11.0 (9.0–12.0)	10.0 (8.0–11.0)	**<0.001**
MELD-3.0 score	17.0 (13.0–21.0)	21.0 (16.0–26.0)	24.0 (18.0–29.5)	20.0 (15.0–26.0)	**<0.001**
CLIF-C OF score	6.0 (6.0–7.0)	7.0 (6.0–8.0)	8.0 (7.0–10.0)	7.0 (6.0–9.0)	**<0.001**
CLIF-C AD score	55.0 (49.9–60.1)	60.1 (54.6–67.1)	65.6 (60.8–73.9)	60.5 (54.3–67.7)	**<0.001**
**Clinical Outcomes**					
In-hospital mortality	8 (4.9%)	6 (3.7%)	30 (18.4%)	44 (9.0%)	**<0.001**
6-month mortality	75 (46.0%)	99 (60.7%)	121 (74.2%)	295 (60.3%)	**<0.001**
12-month mortality	93 (57.1%)	112 (68.7%)	126 (77.3%)	331 (67.7%)	**<0.001**
Overall mortality	95 (58.3%)	122 (74.8%)	128 (78.5%)	345 (70.6%)	**<0.001**
**In-hospital Complications**					
AVB in hospital	22 (13.5%)	11 (6.7%)	23 (14.1%)	56 (11.5%)	0.068
HE during hospital stay	13 (8.0%)	20 (12.3%)	51 (31.3%)	84 (17.2%)	**<0.001**
AKI in hospital	16 (9.8%)	30 (18.4%)	58 (35.6%)	104 (21.3%)	**<0.001**
Hypotension in hospital	7 (4.3%)	9 (5.5%)	29 (17.8%)	45 (9.2%)	**<0.001**
RRT during hospital	1 (0.6%)	3 (1.8%)	12 (7.4%)	16 (3.3%)	0.001
Mechanical ventilation during stay	8 (4.9%)	2 (1.2%)	23 (14.1%)	33 (6.7%)	**<0.001**
Shifted to ICU	39 (23.9%)	38 (23.3%)	74 (45.4%)	151 (30.9%)	**<0.001**
No. of organ failures during stay	1.0 (0.0–2.0)	2.0 (1.0–2.0)	2.0 (1.0–3.0)	2.0 (1.0–2.0)	**<0.001**
DNR status	4 (2.5%)	4 (2.5%)	18 (11.0%)	26 (5.3%)	**<0.001**

An identifiable acute precipitant was recorded in 110 patients (22.5%): bacterial infection or septic shock in 43 (8.8%), sAH in 30 (6.1%), hepatotoxicity from complementary and alternative medicines in 26 (5.3%), conventional drug-induced liver injury in 6 (1.2%) and viral hepatitis flare or reactivation in 7 (1.4%). All 30 patients with sAH had ACLF at admission. The admission pathway, presenting problems, precipitants, level of care and special populations in the study cohort (S1 Table).

The median prognostic scores were: Child–Turcotte–Pugh (CTP) score 10 (IQR 8–11), Model for End-Stage Liver Disease 3.0 (MELD-3.0) score 20 (IQR 15–26), CLIF-C OF score 7 (IQR 6–9), and CLIF-C AD score 60.5 (IQR 54.3–67.7)

Mortality rates were 9.0% (44/489) in-hospital, 60.3% (295/489) at 6 months, 67.7% (331/489) at 12 months, and 70.6% (345/489) overall during follow-up. Median follow-up from admission to death or last contact was 77 days (IQR 6–475) for the whole cohort and 873 days (IQR 497–1149) among patients alive at last contact (reverse Kaplan–Meier estimate 930 days); total follow-up was 533 person-years.

The primary adjusted model (log IL-6, age, sex and MELD-3.0) had approximately 11 events per variable. C-reactive protein was missing in 21 patients (4.3%) and procalcitonin in 52 (10.6%); analyses involving these variables were performed on complete cases, and the denominators are reported with each estimate. All other variables were complete.

### 4.2. Interleukin-6 distribution

Serum IL-6 levels at admission were markedly right-skewed, with a median of 23.8 pg/mL (IQR 9.4–71.8; range 0.1–5500 pg/mL). The distribution was log-normal, and natural logarithm-transformed IL-6 (loge IL-6) was used for parametric analyses. For descriptive purposes, patients were stratified into tertiles: T1 (≤13.1 pg/mL, n = 163), T2 (13.3–50.4 pg/mL, n = 163), and T3 (≥51.2 pg/mL, n = 163). Quartile boundaries were: Q1 (≤9.4), Q2 (9.7–23.8), Q3 (23.9–71.8), and Q4 (≥72.0 pg/mL; n = 122–123 each). (Fig 1; Table 1)

**Fig 1:**
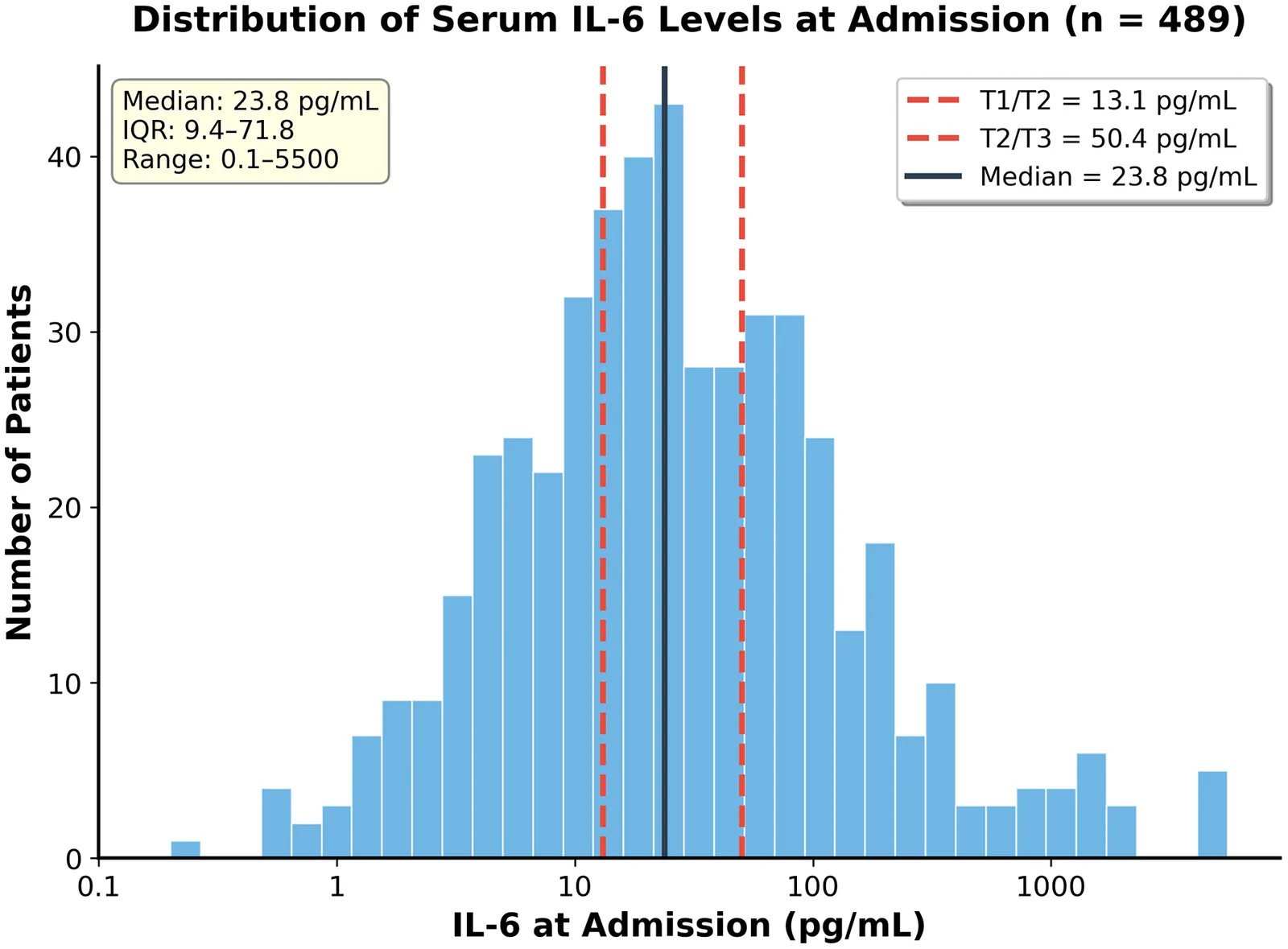
Distribution of serum interleukin-6 (IL-6) levels at admission in 489 hospitalized patients with chronic liver disease. The histogram displays IL-6 values on a logarithmic scale. The solid black line indicates the median (23.8 pg/mL). Dashed red lines denote tertile boundaries (T1/T2 = 13.1 pg/mL; T2/T3 = 50.4 pg/mL). The interquartile range was 9.4–71.8 pg/mL, with values ranging from 0.1 to 5500 pg/mL.

Patients in the highest IL-6 tertile (T3) had significantly more severe disease across virtually all clinical and laboratory parameters compared with T1. While age (p = 0.559) and sex distribution (p = 0.600) were similar across tertiles, clinical syndrome distribution differed significantly (p < 0.05), with a progressive increase in ACLF prevalence from T1 (16.6%) to T3 (27.6%) and a concomitant decrease in compensated cirrhosis from T1 (13.5%) to T3 (4.3%).

ACLF grade showed a striking gradient across IL-6 tertiles (p < 0.001). The proportion of patients with no ACLF decreased from 32.5% (T1) to 14.7% (T3), while ACLF grade 3 increased from 3.7% (T1) to 13.5% (T2) to 29.4% (T3). Similarly, clinical features at admission demonstrated consistent and highly significant associations with IL-6 tertile (all p < 0.001): infection was present in 14.1% (T1) versus 50.3% (T3), HE in 17.2% versus 38.0%, AKI in 10.4% versus 31.9%, hypotension in 2.5% versus 14.1%, and MICU admission in 23.9% versus 44.2%.

Laboratory parameters followed the same trend. Across T1 to T3, median haemoglobin decreased (10.8 → 9.8 g/dL), while total leukocyte count (5.6 → 9.4 × 103/µL), total bilirubin (2.2 → 4.1 mg/dL), INR (1.5 → 2.0), and creatinine (1.0 → 1.3 mg/dL) all increased (all p < 0.001). Albumin declined (3.0 → 2.6 g/dL) and sodium fell (135.5 → 131.0 mEq/L; both p < 0.001). Inflammatory markers showed the most dramatic gradients: CRP rose from 6.0 (T1) to 44.1 mg/L (T3), and procalcitonin from 0.1 to 0.9 ng/mL (both p < 0.001). All composite prognostic scores – CTP (8 → 11), MELD-3.0 (17 → 24), CLIF-C OF (6 → 8), and CLIF-C AD (55.0 → 65.6) – demonstrated significant stepwise increases across IL-6 tertiles (all p < 0.001).

### 4.3. Correlations between IL-6 and clinical variables and organ failure burden

Spearman rank correlation analysis revealed significant associations between IL-6 and multiple clinical and laboratory parameters. The strongest positive correlations were with C-reactive protein (ρ = 0.546, p < 0.001), procalcitonin (ρ = 0.507, p < 0.001), and the CLIF-C AD score (ρ = 0.504, p < 0.001). Among prognostic scoring systems, CTP score (ρ = 0.428), MELD-3.0 (ρ = 0.396), and CLIF-C OF (ρ = 0.382) all correlated significantly with IL-6 (all p < 0.001). Albumin (ρ = −0.318) and sodium (ρ = −0.274) were inversely correlated with IL-6. IL-6 levels were significantly elevated in patients with adverse clinical features at admission (Table 2; S1 Fig).

**Table 2:** IL-6 Levels by Binary Clinical Variables (Mann-Whitney U Test).

Variable	Present: Median (IQR)	N- present	Absent: Median (IQR)	N – absent	p-value
Diabetes	21.2 (7.1–63.1)	298	28.6 (11.7–110.1)	191	0.003
Hypertension	30.4 (9.9–72.0)	88	22.9 (9.4–71.2)	401	0.391
Obesity	15.8 (5.3–37.2)	154	29.7 (11.9–83.5)	335	**<0.001**
MICU admission	47.9 (12.9–107.9)	150	19.0 (7.8–54.4)	339	**<0.001**
AVB at admission	20.4 (5.2–75.0)	91	24.8 (10.1–71.1)	398	0.166
HE at admission	46.1 (17.3–109.5)	131	18.9 (7.5–57.2)	358	**<0.001**
AKI at admission	52.7 (19.8–125.8)	103	20.4 (7.3–63.4)	386	**<0.001**
Hypotension at admission	108.0 (54.4–345.0)	31	22.1 (8.5–64.9)	458	**<0.001**
Infection present	63.1 (21.6–199.8)	145	17.0 (6.8–46.1)	344	**<0.001**
Ascites at admission	32.7 (13.9–73.5)	267	15.5 (5.9–63.6)	222	**<0.001**
Refractory ascites	45.6 (20.3–103.2)	132	18.5 (6.7–63.5)	357	**<0.001**
Hyponatremia	30.9 (13.6–75.0)	178	20.7 (6.9–66.9)	311	0.002
HCC diagnosed	21.0 (9.8–72.4)	97	24.1 (9.3–71.0)	392	0.782
RRT at admission	74.6 (71.5–206.5)	9	22.9 (9.0–70.5)	480	0.002
Mechanical ventilation at admission	197.1 (69.8–453.9)	15	22.9 (8.9–69.7)	474	**<0.001**

The most pronounced differences were observed with infection (63.1 vs. 17.0 pg/mL, p < 0.001), mechanical ventilation (197.1 vs. 22.9, p < 0.001), and hypotension (108.0 vs. 22.1, p < 0.001). IL-6 demonstrated a striking dose-response relationship with the number of organ failures at admission (Kruskal–Wallis p < 0.001; Spearman ρ = 0.390, p < 0.001) (S2 Fig). Median IL-6 levels rose progressively from 13.0 pg/mL (IQR 4.3–36.6) in patients with zero organ failures to 1091.0 (526.4–1700.7) with five organ failures. A similar gradient was observed during the hospital course (ρ = 0.382, p < 0.001).

### 4.4. IL-6 by infection site and clinical syndrome

Site categories in this paragraph are mutually exclusive, as recorded at admission. Among patients with documented infection (n = 145), the highest IL-6 levels were observed in bloodstream infections (n = 59; median 96.4, IQR 36.5–352.4 pg/mL), followed by combined blood and urinary infections (n = 9; 29.7, 23.0–202.7). A harmonised any-site tabulation, in which infections at more than one site contribute to each category, together with site-specific IL-6 levels and in-hospital mortality, is provided in S2 Table. IL-6 levels also varied significantly across clinical syndromes (Fig 2). Median levels were lowest in compensated cirrhosis (11.3, IQR 4.0–22.1 pg/mL, n = 39), intermediate in decompensated cirrhosis (22.1, 8.7–68.5, n = 338), and highest in ACLF (36.5, 16.5–114.4, n = 112). All pairwise comparisons were significant (compensated vs. decompensated, p = 0.001; compensated vs. ACLF, p < 0.001; decompensated vs. ACLF, p = 0.003).

**Fig 2:**
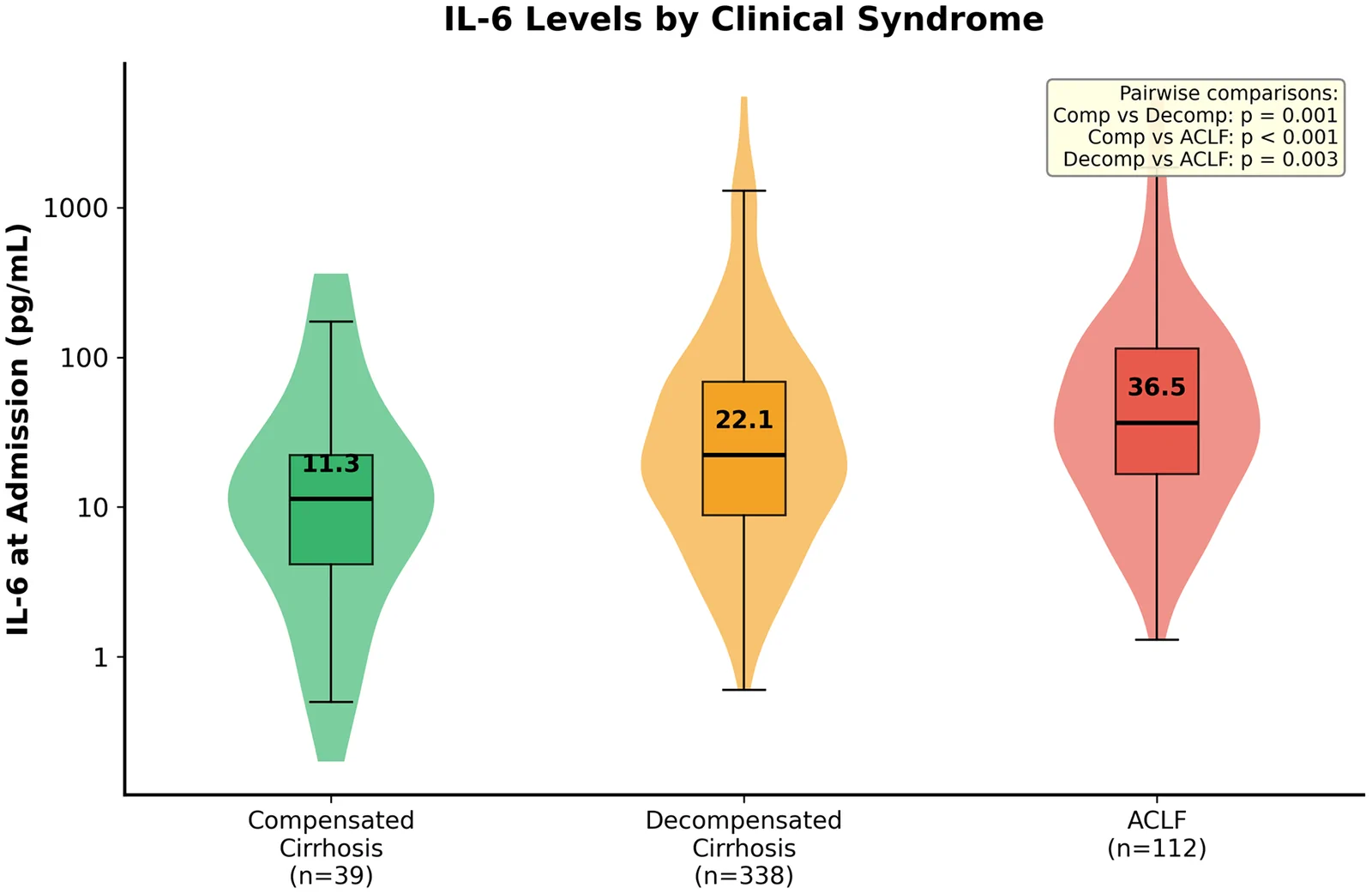
Serum IL-6 levels at admission by clinical syndrome. Violin plots with embedded box plots show the distribution of IL-6 (logarithmic scale) across compensated cirrhosis (n = 39; median 11.3 pg/mL), decompensated cirrhosis (n = 338; median 22.1 pg/mL), and acute-on-chronic liver failure (ACLF; n = 112; median 36.5 pg/mL). Pairwise Mann–Whitney U comparisons: compensated vs. decompensated, p = 0.001; compensated vs. ACLF, p < 0.001; decompensated vs. ACLF, p = 0.003.

### 4.5. IL-6 and mortality outcomes

IL-6 levels at admission were significantly higher in non-survivors compared with survivors across all mortality endpoints (Fig 3A). For in-hospital mortality, non-survivors had a median IL-6 of 66.9 pg/mL (IQR 43.9–201.5) versus 21.6 (7.9–67.1) in survivors (p < 0.001). For 6-month mortality, corresponding values were 35.5 (12.9–88.7) versus 14.3 (6.2–36.9, p < 0.001); for 12-month mortality, 29.7 (11.5–81.7) versus 14.9 (5.9–45.8, p < 0.001); and for overall mortality, 28.6 (11.8–75.4) versus 13.7 (5.3–46.8, p < 0.001).

**Fig 3:**
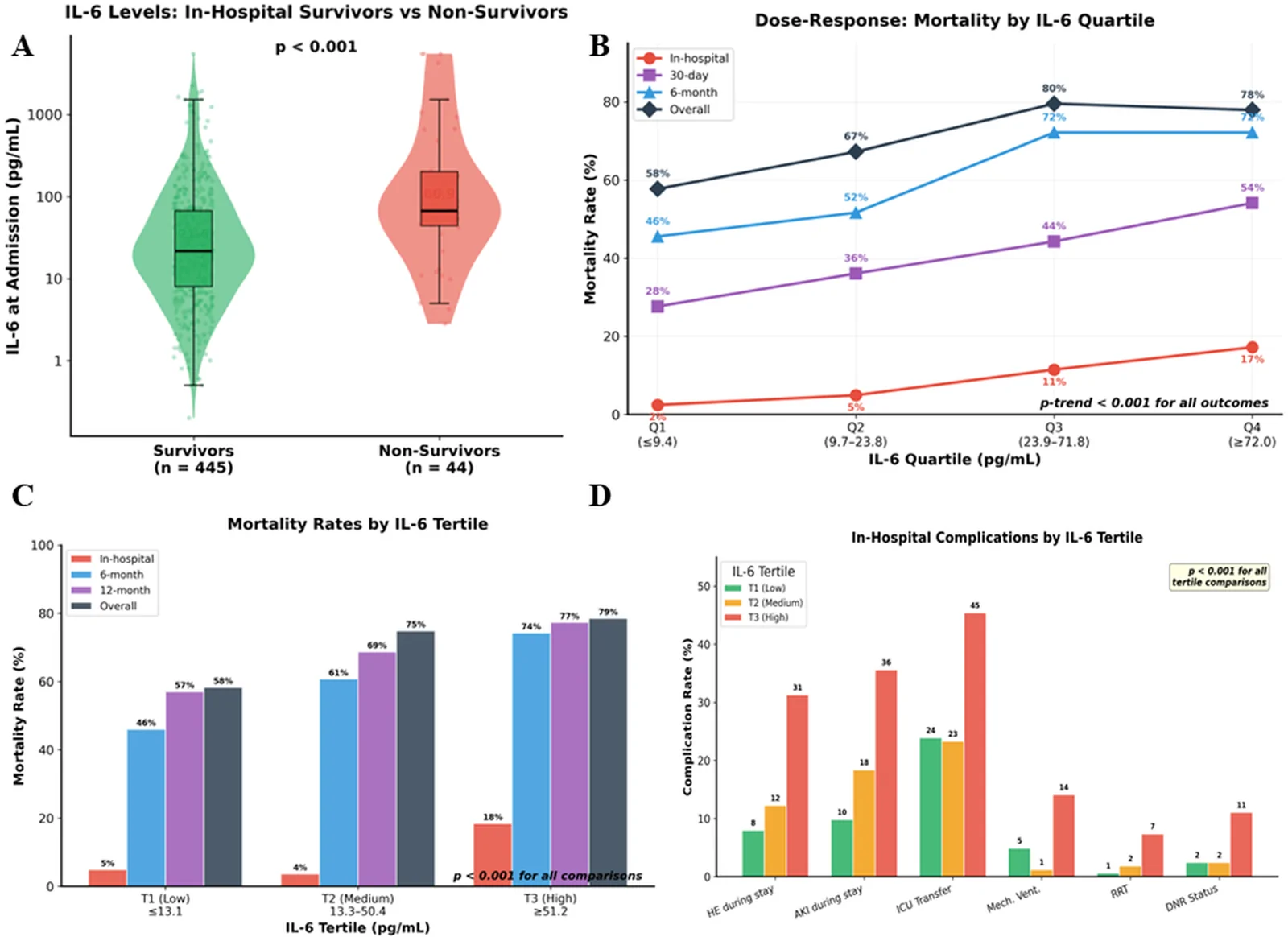
Association of IL-6 with mortality and in-hospital complications. **(A)** Violin plots with individual data points comparing IL-6 levels between in-hospital survivors (n = 445) and non-survivors (n = 44); p < 0.001, Mann–Whitney U test. **(B)** Dose–response relationship between IL-6 quartiles (Q1, ≤ 9.4; Q2, 9.7–23.8; Q3, 23.9–71.8; Q4, ≥ 72.0 pg/mL) and mortality rates at four endpoints (in-hospital, 30-day, 6-month, and overall); p-trend < 0.001 for all outcomes. **(C)** Mortality rates by IL-6 tertile (T1, ≤ 13.1; T2, 13.3–50.4; T3, ≥ 51.2 pg/mL) for in-hospital, 6-month, 12-month, and overall mortality; p < 0.001 for all comparisons. **(D)** Rates of in-hospital complications by IL-6 tertile, including hepatic encephalopathy (HE), acute kidney injury (AKI), ICU transfer, mechanical ventilation, renal replacement therapy (RRT), and do-not-resuscitate (DNR) status; p < 0.001 for all tertile comparisons.

A significant dose-response relationship was observed between IL-6 quartiles and mortality at all time points (p-trend < 0.001 for all) (Fig 3B). In-hospital mortality rose from 2.4% (Q1) to 17.2% (Q4). Six-month mortality increased from 45.5% to 72.1%, and overall mortality from 57.7% to 77.9% across Q1 to Q4. Linearity testing confirmed that a log-linear model was adequate for in-hospital (p = 0.985 for quadratic term) and 6-month mortality (p = 0.129), although mild non-linearity was detected for overall mortality (p = 0.04 for quadratic term).

All mortality rates differed significantly across IL-6 tertiles (all p < 0.001) (Fig 3C). In-hospital mortality was 4.9% (T1), 3.7% (T2), and 18.4% (T3). Six-month mortality was 46.0%, 60.7%, and 74.2%, respectively. Overall mortality was 58.3%, 74.8%, and 78.5%. In-hospital complication rates followed the same gradient: overt HE during stay (8.0% vs. 31.3%), AKI during hospitalization (9.8% vs. 35.6%), ICU transfer (23.9% vs. 45.4%), mechanical ventilation (4.9% vs. 14.1%), renal replacement therapy (0.6% vs. 7.4%), and DNR status (2.5% vs. 11.0%) all increased sharply from T1 to T3 (all p < 0.001) (Fig 3D).

### 4.6. Time-dependent analysis

Among the 44 in-hospital deaths, 24 (54.5%) occurred within 7 days (“early deaths”) and 20 (45.5%) after 7 days. Median IL-6 levels were similarly elevated in early deaths (69.5, IQR 37.8–184.6 pg/mL) and late deaths (65.2, 43.9–269.2; p = 0.814 between groups), both significantly higher than survivors (21.6, 7.9–67.1; both p ≤ 0.001). Landmark analyses at 30 and 90 days confirmed significant IL-6 associations: 30-day mortality (n = 198 events; OR 1.34, 95% CI 1.18–1.51; AUC 0.622) and 90-day mortality (n = 251 events; OR 1.44, 1.26–1.63; AUC 0.656).

### 4.7. Logistic regression and discriminative performance

In univariate logistic regression, IL-6 was a significant predictor of all mortality endpoints: in-hospital mortality (OR 1.63, 95% CI 1.35–1.96, p < 0.001), 6-month mortality (OR 1.40, 1.23–1.59, p < 0.001), 12-month mortality (OR 1.31, 1.15–1.50, p < 0.001), and overall mortality (OR 1.34, 1.16–1.54, p < 0.001). After adjustment for age, sex, and MELD-3.0, IL-6 remained an independent predictor of in-hospital mortality (adjusted OR 1.34, 95% CI 1.08–1.67, p = 0.009). However, for longer-term outcomes, IL-6’s effect was attenuated by MELD-3.0 adjustment: 6-month mortality (OR 1.13, 0.98–1.31, p = 0.091), 12-month mortality (OR 1.06, 0.92–1.23, p = 0.422), and overall mortality (OR 1.09, 0.94–1.27, p = 0.247) [Table 3]. In a comprehensive multivariate logistic regression model (n = 468, 43 events), the independent predictors of in-hospital mortality were MELD-3.0 (OR 1.08, 95% CI 1.03–1.14, p < 0.001), overt HE at admission (OR 2.35, 1.13–4.87, p = 0.022), and log IL-6 (OR 1.21, 0.94–1.54, p = 0.133). Log IL-6 and CRP were moderately correlated (ρ = 0.546). Variance inflation factor analysis confirmed low multicollinearity for log IL-6 (VIF 1.58) and CRP (VIF 1.34), while MELD-3.0 (VIF 6.14), CLIF-C OF (VIF 3.54), and CLIF-C AD (VIF 3.56) showed higher collinearity. Receiver operating characteristic analysis demonstrated that IL-6 had the highest discriminative performance for in-hospital mortality (AUC 0.715, 95% CI 0.631–0.793), with decreasing performance for longer-term outcomes (6-month AUC 0.647, 0.599–0.696; 12-month AUC 0.617, 0.562–0.666; overall AUC 0.626, 0.572–0.680) (Fig 4).

**Table 3:** Logistic Regression Of IL-6 For Predicting Mortality Outcomes.

Outcome	Model	OR	95% CI	p-value
In-hospital mortality	Univariate (log IL-6)	1.63	1.35–1.96	<0.001
In-hospital mortality	Adjusted (Age, Sex, MELD-3.0)	1.34	1.08–1.67	0.009
6-month mortality	Univariate (log IL-6)	1.4	1.23–1.59	<0.001
6-month mortality	Adjusted (Age, Sex, MELD-3.0)	1.13	0.98–1.31	0.091
12-month mortality	Univariate (log IL-6)	1.31	1.15–1.50	<0.001
12-month mortality	Adjusted (Age, Sex, MELD-3.0)	1.06	0.92–1.23	0.422
Overall mortality	Univariate (log IL-6)	1.34	1.16–1.54	<0.001
Overall mortality	Adjusted (Age, Sex, MELD-3.0)	1.09	0.94–1.27	0.247

**Fig 4:**
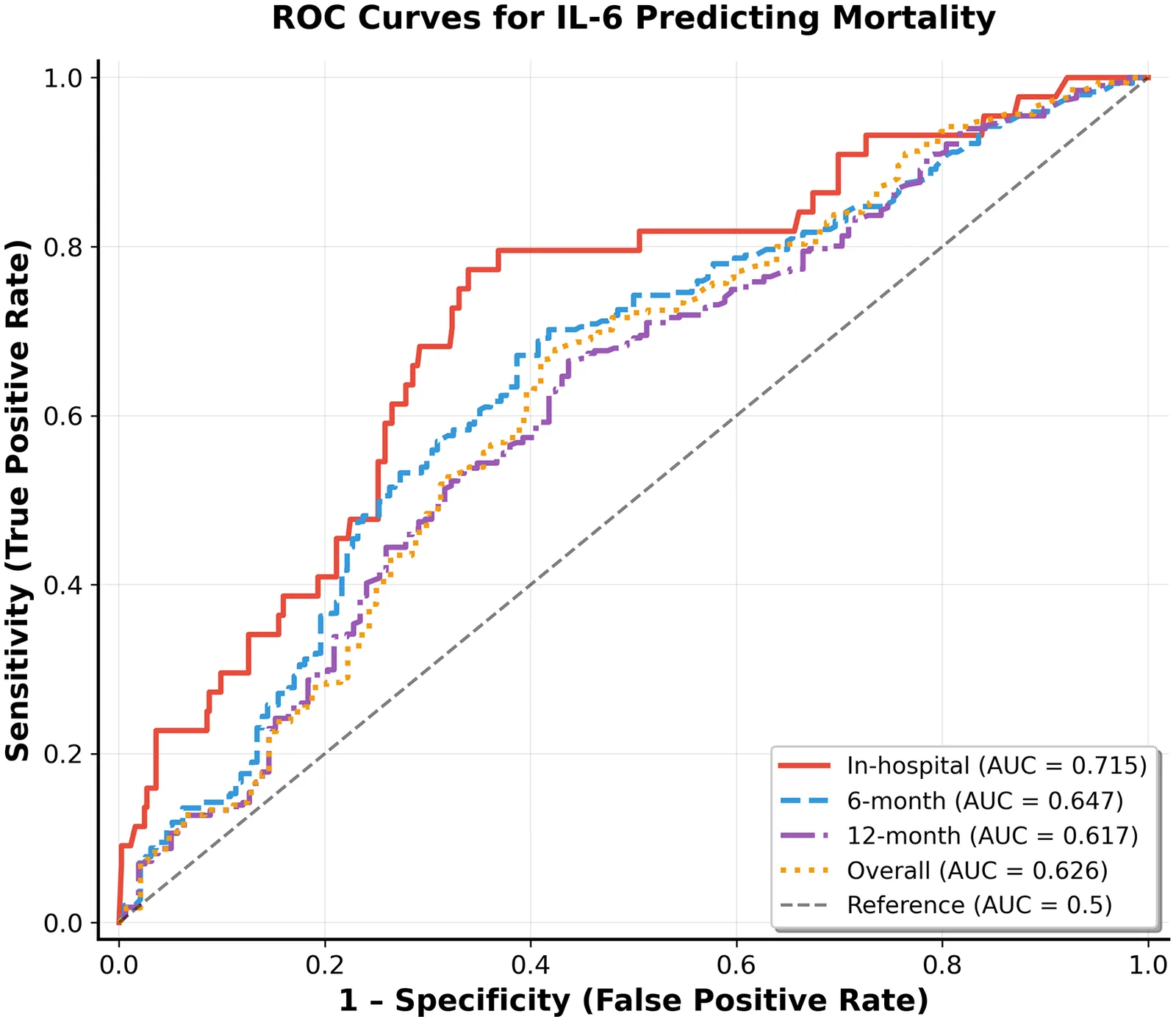
Receiver operating characteristic (ROC) curves for serum IL-6 at admission as a predictor of mortality at multiple time points. Area under the curve (AUC): in-hospital, 0.715; 6-month, 0.647; 12-month, 0.617; overall, 0.626. The dashed grey diagonal represents the reference line (AUC = 0.5).

The optimal IL-6 cutoff for in-hospital mortality by Youden’s index was 41.6 pg/mL (sensitivity 77.3%, specificity 66.1%, S3 Fig). At a specificity threshold of ≥80%, the corresponding cutoff was 87.3 pg/mL (sensitivity 40.9%). For 6-month mortality, the optimal cutoff was 19.2 pg/mL (sensitivity 67.1%, specificity 61.3%). Analysis at predefined cutoffs revealed that IL-6 ≥ 10 pg/mL achieved 90.9% sensitivity for in-hospital mortality with a negative predictive value (NPV) of 96.9%.

### 4.8. Comparative and incremental predictive value

For in-hospital mortality, IL-6 alone (AUC 0.715) performed comparably to CTP (0.681), CRP (0.688), and procalcitonin (0.698), but was outperformed by MELD-3.0 (0.762), CLIF-C OF (0.774), and CLIF-C AD (0.763) (Fig 5). The novel IL-6/albumin ratio (0.723) slightly outperformed IL-6 alone. Among combined models, CLIF-C OF + IL-6 achieved the highest AUC (0.802), followed by MELD + IL-6 (0.785) and MELD + CRP (0.779).

**Fig 5:**
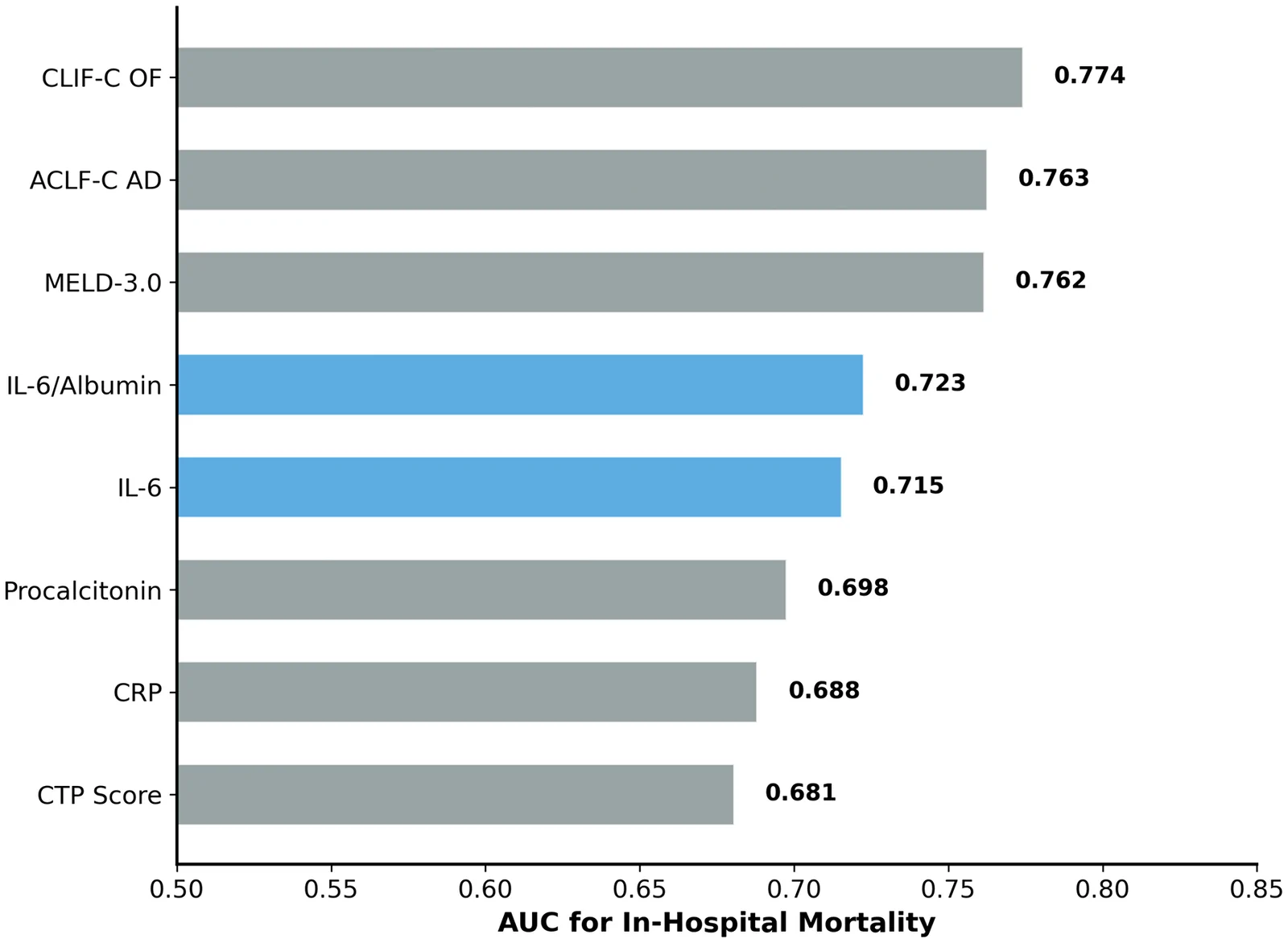
Comparative discriminative performance (AUC) for in-hospital mortality across prognostic scores, inflammatory biomarkers, and IL-6-derived indices. CLIF-C OF, Chronic Liver Failure Consortium Organ Failure score; CLIF-C AD, Chronic Liver Failure-Consortium Acute Decompensation score; MELD-3.0, Model for End-Stage Liver Disease 3.0; IL-6/Albumin, IL-6-to-albumin ratio; CRP, C-reactive protein; CTP, Child–Turcotte–Pugh.

Adding IL-6 to MELD-3.0 significantly improved model fit for in-hospital mortality prediction (likelihood ratio test p = 0.008; ΔAUC = +0.023; integrated discrimination improvement [IDI] = 0.0225) (Table 4). The incremental gain from IL-6 was comparable to that from CRP (ΔAUC = +0.025). For 6-month mortality, the improvement was smaller but statistically significant (p = 0.022; ΔAUC = +0.009). For 12-month (p = 0.188) and overall mortality (p = 0.101), the incremental value was not statistically significant.

**Table 4:** Incremental Predictive Value of IL-6 Over MELD-3.0.

Outcome	AUC (MELD alone)	AUC (MELD + IL-6)	ΔAUC	IDI	LR Test p-value	AUC (MELD + CRP)
In-hospital mortality	0.762	0.785	+0.023	0.0225	0.008	0.779
6-month mortality	0.712	0.721	+0.009	0.0102	0.022	0.724
12-month mortality	0.703	0.705	+0.002	0.0036	0.188	0.702
Overall mortality	0.701	0.705	+0.004	0.0060	0.101	0.697

Internal validation using 500 bootstrap replications yielded an optimism-corrected C-statistic of 0.774 (apparent 0.787, mean optimism 0.014) for the multivariate model (log IL-6 + age + MELD-3.0) predicting in-hospital mortality, confirming model stability. The Hosmer–Lemeshow goodness-of-fit test indicated adequate calibration of the IL-6 logistic model (χ² = 11.47, df = 8, p = 0.176).

### 4.9. Survival analysis

In univariate Cox regression, log IL-6 was significantly associated with overall mortality (HR 1.21, 95% CI 1.14–1.30, p < 0.001; concordance index 0.60). In a multivariate Cox model (n = 468, 336 events; concordance 0.68), the independent predictors of overall mortality were MELD-3.0 (HR 1.07, 1.05–1.08, p < 0.001), age (HR 1.02, 1.01–1.03, p < 0.001), and HE at admission (HR 1.36, 1.06–1.74, p = 0.02). Log IL-6 was not independently significant in this model (HR 1.05, 0.97–1.13, p = 0.28). In a separate Cox model for 6-month mortality, log IL-6 retained independent significance (HR 1.10, 95% CI 1.02–1.19, p = 0.019) alongside MELD-3.0 (HR 1.07, 1.06–1.09, p < 0.001) and age (HR 1.02, 1.01–1.04, p < 0.001).

Kaplan–Meier survival analysis demonstrated marked separation of survival curves by IL-6 tertile (log-rank χ² = 25.54, p < 0.001) (Fig 6). Median survival decreased steeply with rising IL-6: 230 days (T1), 71 days (T2), and 27 days (T3). The difference was most pronounced in the first 90 days, with subsequent convergence of curves.

**Fig 6:**
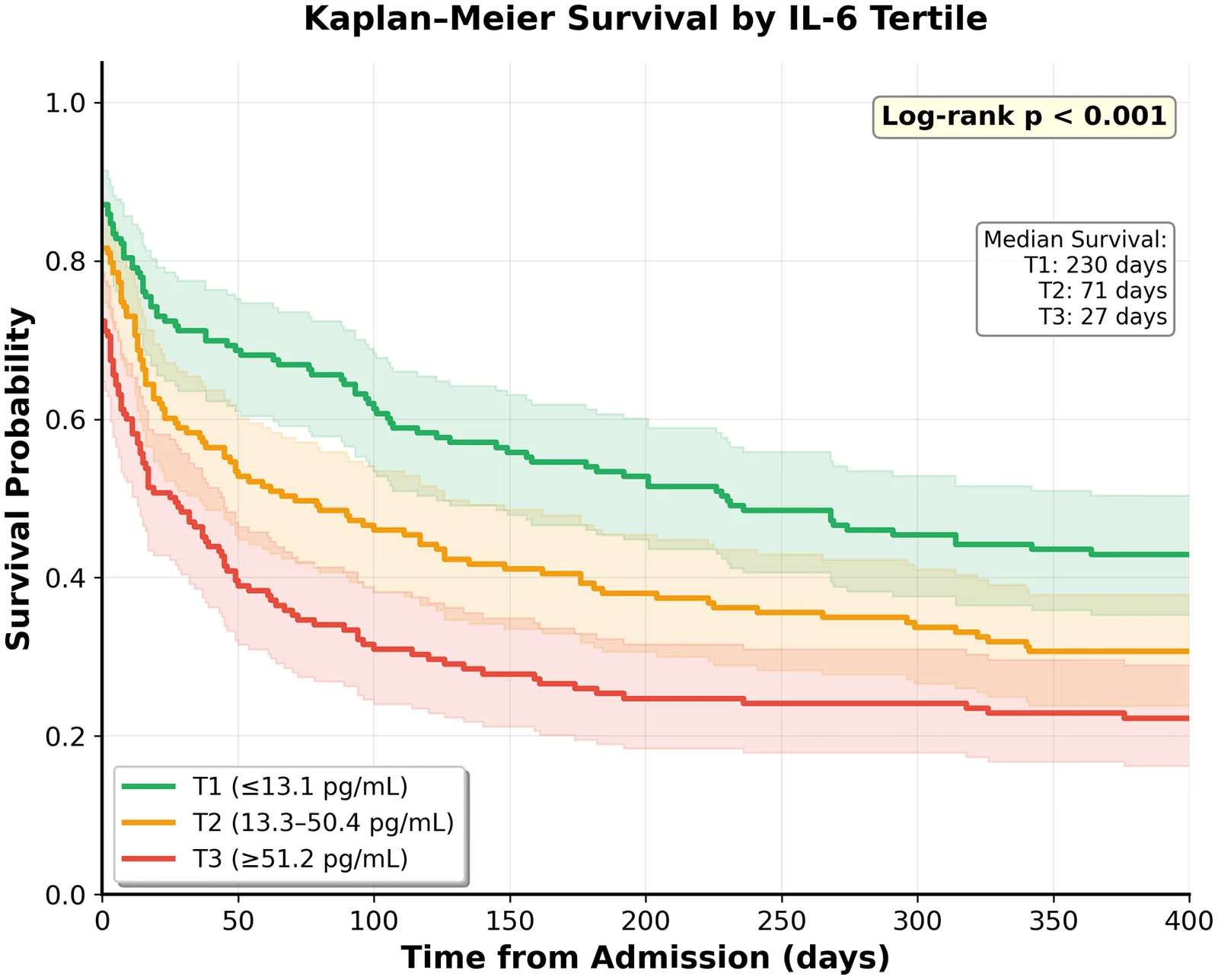
Kaplan–Meier survival curves stratified by IL-6 tertile (T1, ≤ 13.1; T2, 13.3–50.4; T3, ≥ 51.2 pg/mL). Shaded bands represent 95% confidence intervals. Median survival: T1, 230 days; T2, 71 days; T3, 27 days. Log-rank p < 0.001.

### 4.10. Subgroup and interaction analyses

The discriminative performance of IL-6 for in-hospital mortality varied substantially across clinical subgroups (Fig 7). The highest AUC was observed in patients with ALD etiology (AUC 0.802; OR 2.21, 95% CI 1.60–3.06, S4 Fig), substantially exceeding that in MASLD patients (AUC 0.648; OR 1.27, 0.92–1.74). Among patients with ACLF, IL-6 demonstrated strong discrimination (AUC 0.720; OR 1.65, 1.35–2.02), while in patients without ACLF, too few events precluded analysis. IL-6 discriminated better among MICU patients (AUC 0.693; OR 1.61) than ward patients (AUC 0.609; OR 1.25, non-significant), and similarly among patients with HE (AUC 0.692) versus without HE (AUC 0.684).

**Fig 7:**
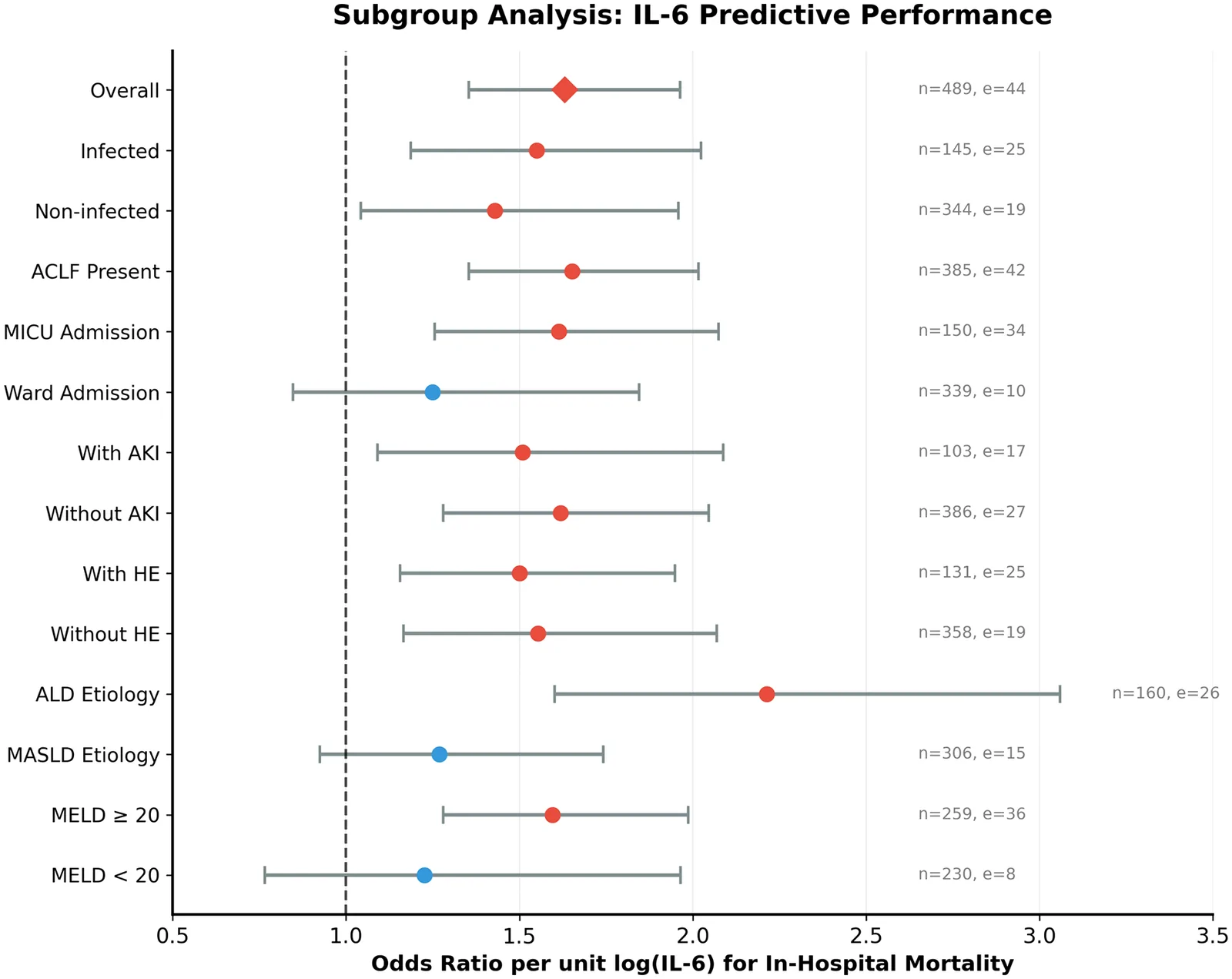
Subgroup analysis of IL-6 predictive performance for in-hospital mortality. Forest plot displays odds ratios (ORs) per unit increase in log-transformed IL-6 with 95% confidence intervals across clinically defined subgroups. Red circles indicate point estimates with confidence intervals excluding the null; blue circles indicate confidence intervals crossing the null. Sample sizes (n) and number of events (e) are shown for each subgroup. The overall OR (diamond) is shown for reference.

Formal interaction testing identified one significant effect modification (Table 5): the IL-6 × ALD etiology interaction (interaction OR 1.93, 95% CI 1.25–2.99, p = 0.002), indicating that the prognostic impact of IL-6 is significantly amplified in ALD compared with other etiologies. A borderline interaction was observed with ACLF (interaction OR 2.42, 0.75–7.77, p = 0.083). No significant interactions were found with infection, AKI, overt HE, MICU admission, diabetes, or MELD ≥20.

**Table 5:** Interaction Analysis – IL-6 × Clinical Variable Interactions for In-Hospital Mortality.

Interaction Term	Interaction OR	95% CI	p-interaction	Significant
IL-6 × Infection	1.08	0.72–1.64	0.699	No
IL-6 × ACLF (any)	2.42	0.75–7.77	0.083	No (trend)
IL-6 × AKI	0.93	0.62–1.39	0.732	No
IL-6 × HE	0.97	0.66–1.42	0.863	No
IL-6 × MICU	1.29	0.81–2.05	0.270	No
IL-6 × Diabetes	0.87	0.59–1.28	0.463	No
IL-6 × MELD ≥ 20	1.30	0.77–2.19	0.312	No
IL-6 × ALD etiology	1.93	1.25–2.99	0.002	**Yes**

When stratified by MELD-3.0 tertiles (Fig 8), high IL-6 (≥ median) was associated with significantly worse 6-month (53.6% vs. 32.7%, p = 0.016) and overall mortality (64.3% vs. 45.8%, p = 0.038) among low-MELD patients (score 6–16). In medium-MELD patients (17–24), high IL-6 was associated with higher in-hospital mortality (8.1% vs. 0%, p = 0.015) and 6-month mortality (71.6% vs. 53.6%, p = 0.025). In high-MELD patients (≥25), IL-6 did not significantly differentiate outcomes (in-hospital 23.3% vs. 12.8%, p = 0.243; overall 87.1% vs. 82.1%, p = 0.609).

**Fig 8:**
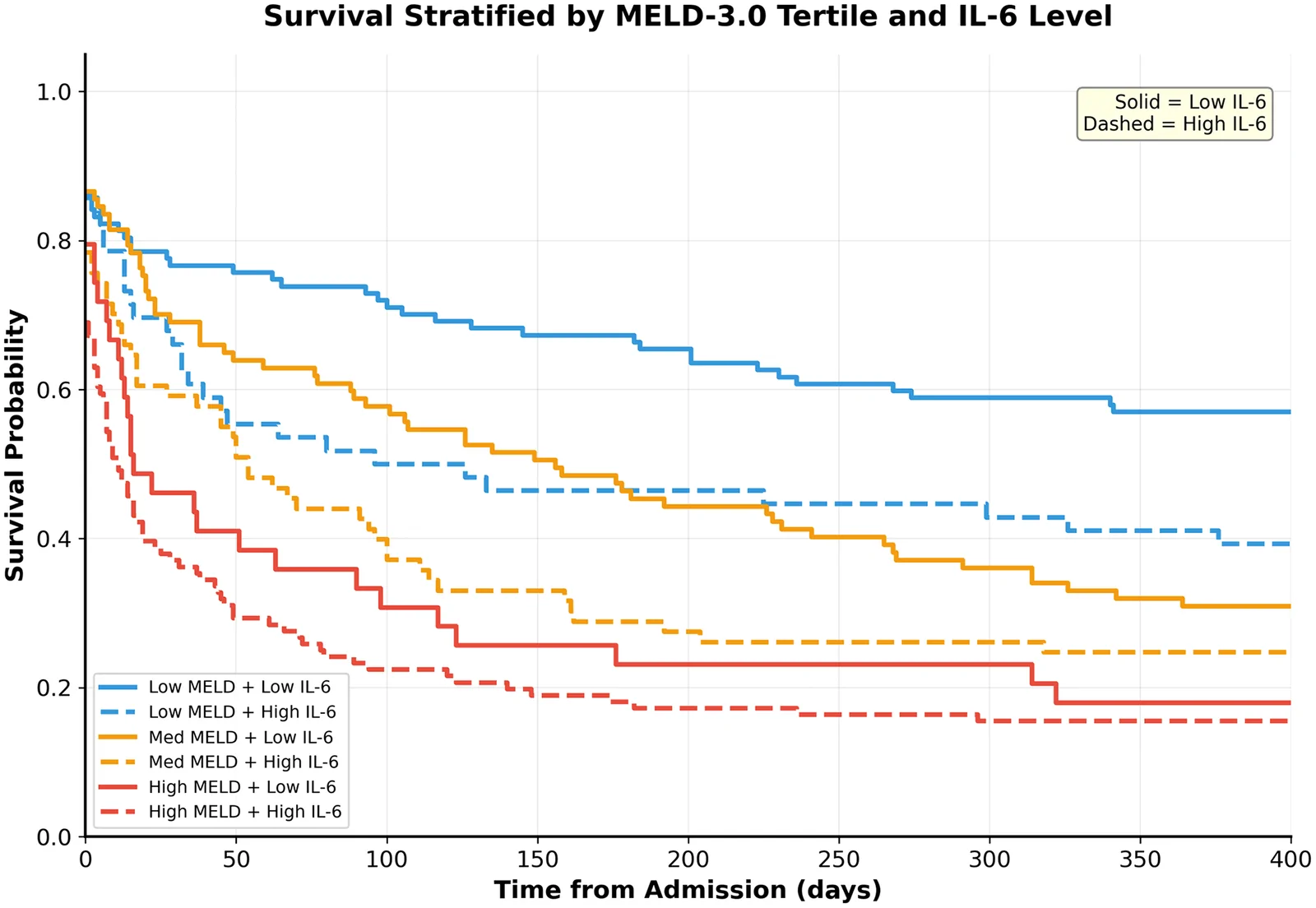
Kaplan–Meier survival curves stratified by combined MELD-3.0 tertile (low, medium, high) and IL-6 level (low vs. high, dichotomized at the median). Solid lines represent patients with low IL-6; dashed lines represent patients with high IL-6. Within each MELD stratum, high IL-6 was associated with worse survival.

### 4.11. Mediation analysis

Mediation analysis using the Baron and Kenny approach revealed that infection partially mediated the IL-6–mortality relationship (Table 6). The total effect of log IL-6 on in-hospital mortality (OR 1.63, 1.35–1.96) was reduced to a direct effect of OR 1.50 (1.22–1.83) after controlling for infection, with 17.3% of the total effect mediated through the infection pathway. IL-6 was itself a strong predictor of infection (OR 1.76, 1.52–2.04, p < 0.001). In contrast, CRP mediated only 9.3% of IL-6’s mortality effect.

**Table 6:** Mediation Analysis – Infection and CRP as Mediators of IL-6 on In-Hospital Mortality.

Path	OR (95% CI)	p-value	Interpretation
			**Mediator: Infection**
Path c (Total: IL-6 → Mortality)	1.63 (1.35–1.96)	<0.001	Total effect of IL-6
Path a (IL-6 → Infection)	1.76 (1.52–2.04)	<0.001	IL-6 predicts infection
Path c’ (Direct: IL-6 → Mortality | Infection)	1.50 (1.22–1.83)	<0.001	Attenuated but significant
Proportion mediated	17.3%	—	Partial mediation
			**Mediator: CRP**
Path c (Total: IL-6 → Mortality)	1.63 (1.35–1.96)	<0.001	Total effect
Path c’ (Direct: IL-6 → Mortality | CRP)	1.56	<0.001	Minimal attenuation
Proportion mediated by CRP	9.3%	—	Modest mediation

### 4.12. IL-6 and clinical decision endpoints

Admission IL-6 levels were significantly associated with key clinical decision markers. Patients who transitioned to DNR (do-not-resuscitate) status had markedly higher IL-6 (71.8, IQR 46.3–192.1 pg/mL) than those who did not (22.2, 8.5–68.1; p < 0.001). Patients who were subsequently transferred to the ICU during hospitalization also had higher admission IL-6 (48.9, 13.0–107.9 vs. 18.9, 7.8–52.7; p < 0.001). Among survivors, IL-6 correlated weakly but significantly with length of hospital stay (Spearman ρ = 0.180, p < 0.001), with T3 patients having a longer median stay (6 days, IQR 4–9) compared to T1 (5 days, 3–6).

### 4.13. IL-6/albumin ratio as a novel composite biomarker

The IL-6/albumin ratio, combining the inflammatory signal of IL-6 with the synthetic function marker albumin, showed improved discrimination compared with IL-6 alone. For in-hospital mortality, the ratio yielded an AUC of 0.723 versus 0.715 for IL-6 alone, with clear separation between survivors (median 8.0) and non-survivors (28.8, p < 0.001). For 6-month mortality, corresponding values were AUC 0.658 versus 0.646 (alive 4.7 vs. dead 14.2, p < 0.001), and for overall mortality, AUC 0.641 versus 0.625 (alive 4.2 vs. dead 11.6, p < 0.001).

### 4.14. Sensitivity analyses

To assess the robustness of findings, sensitivity analyses were performed after excluding 5 patients with extreme IL-6 values (≥99th percentile, > 2495.6 pg/mL). Results were consistent: in-hospital mortality OR 1.51 (95% CI 1.23–1.86, AUC 0.689), 6-month mortality OR 1.42 (1.24–1.62, AUC 0.646), and overall mortality OR 1.36 (1.18–1.58, AUC 0.626). Estimates were similar to those obtained in the full cohort.

### 4.15 Bayesian Inference

Bayesian logistic regression with weakly informative priors (Normal[0,1] on standardized coefficients) was fitted using Hamiltonian Monte Carlo (NUTS sampler; 2000 posterior draws after 1000 tuning iterations per chain) (Fig 9A, Fig 9B). After adjustment for age, sex, and MELD-3.0, the Bayesian posterior distributions for log IL-6 revealed a gradient of evidence strength across mortality time points:

- In-hospital mortality: Posterior OR 1.33 (95% credible interval [CrI] 1.08–1.67), with P(β > 0 | data) = 99.6%, constituting very strong evidence of a positive association.
- 6-month mortality: Posterior OR 1.13 (95% CrI 0.98–1.31), P(β > 0 | data) = 95.6%, representing strong evidence.
- Overall mortality: Posterior OR 1.10 (95% CrI 0.94–1.28), P(β > 0 | data) = 88.8%, representing moderate evidence.

**Fig 9:**
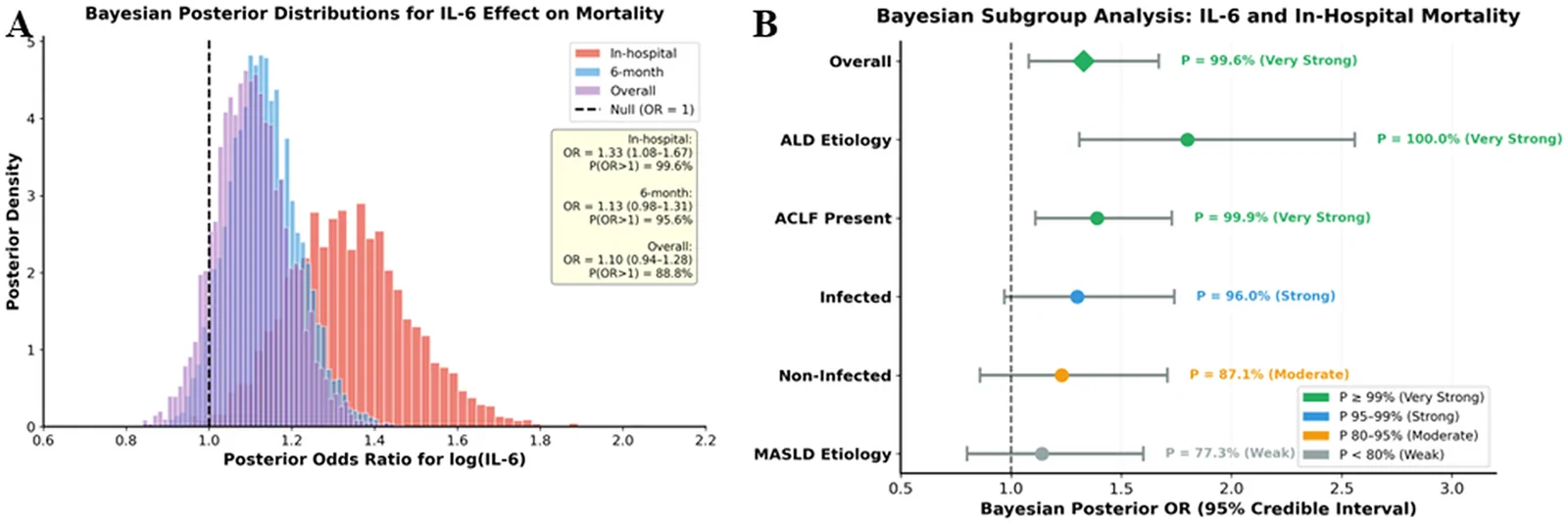
Bayesian analysis of IL-6 and mortality. **(A)** Posterior distributions of the odds ratio for log-transformed IL-6 in multivariable logistic regression models for in-hospital mortality (posterior OR 1.33, 95% credible interval [CrI] 1.08–1.67; P(OR > 1) = 99.6%), 6-month mortality (OR 1.13, 95% CrI 0.98–1.31; P(OR > 1) = 95.6%), and overall mortality (OR 1.10, 95% CrI 0.94–1.28; P(OR > 1) = 88.8%). The dashed black line indicates the null value (OR = 1). **(B)** Bayesian subgroup analysis for in-hospital mortality displaying posterior ORs with 95% CrIs and posterior probabilities of a positive association. Evidence strength is color-coded: green, very strong (P ≥ 99%); blue, strong (P 95–99%); orange, moderate (P 80–95%); grey, weak (P < 80%).

Bayesian model comparison using the Widely Applicable Information Criterion (WAIC) and leave-one-out cross-validation (LOO-CV) favoured the model including IL-6 over the model without IL-6 (ΔWAIC = +2.37; ΔLOO = +2.36). Although the magnitude of improvement was modest, the direction consistently supported inclusion of IL-6, and formal model ranking placed the IL-6-inclusive model first.

Bayesian inference stratified by clinical subgroups (Table 7) revealed marked heterogeneity in IL-6’s prognostic signal. The strongest evidence was in ALD patients (posterior OR 1.80, 95% CrI 1.31–2.56; P[β > 0] = 100%) and ACLF patients (OR 1.39, CrI 1.11–1.73; P[β > 0] = 99.9%). In infected patients, evidence was strong (OR 1.30, P = 96.0%), while in non-infected patients it was moderate (OR 1.23, P = 87.1%). MASLD patients showed the weakest evidence (OR 1.14, CrI 0.80–1.60; P[β > 0] = 77.3%).

**Table 7:** Bayesian Subgroup Analysis – Posterior IL-6 Effect on In-Hospital Mortality.

Subgroup	n	Events	Posterior OR	95% CrI	P(β > 0 | data)	Evidence
Overall	489	44	1.33	1.08–1.67	99.6%	Very Strong
Infected	145	25	1.30	0.97–1.74	96.0%	Strong
Non-infected	344	19	1.23	0.86–1.71	87.1%	Moderate
ACLF present	385	42	1.39	1.11–1.73	99.9%	Very Strong
ALD etiology	160	26	1.80	1.31–2.56	100.0%	Very Strong
MASLD etiology	306	15	1.14	0.80–1.60	77.3%	Weak

A classification tree (maximum depth 3, minimum leaf size 20) for in-hospital mortality identified MELD-3.0 as the dominant splitting variable (feature importance 0.726) with IL-6 as the second most important feature (0.274). CTP and CLIF-C OF scores did not contribute additional splits beyond what MELD-3.0 and IL-6 provided.

### 4.16. IL-6 in clinically enriched sub-cohorts: Infection, severe alcohol-associated hepatitis and etiology

IL-6 was examined separately in patients with documented infection at admission, in patients with sAH, in their combination, and within etiology strata. Among the 145 patients with infection (25 in-hospital deaths, 17.2%), IL-6 discriminated in-hospital death (AUC 0.700, 95% CI 0.595–0.805; unadjusted OR per log-unit 1.54, 1.18–2.00), with attenuation after MELD-3.0 adjustment (OR 1.30, 0.97–1.74; p = 0.075). In this stratum the AUC of IL-6 exceeded that of CRP (0.606) and procalcitonin (0.612) but not that of MELD-3.0 (0.732) or CLIF-C OF (0.749). Among the 344 non-infected patients (19 deaths, 5.5%), discrimination was weaker (AUC 0.627, 0.490–0.763; MELD-adjusted OR 1.24, 0.88–1.75). Adding IL-6 to MELD-3.0 increased the AUC by +0.032 in infected patients (likelihood-ratio test p = 0.071) and by +0.003 in non-infected patients (p = 0.23). The cut-off of 41.6 pg/mL had a sensitivity of 92.0% and a negative predictive value of 96.2% in infected patients, but a sensitivity of 57.9% in non-infected patients (S3 Table).

Thirty patients had sAH as the precipitating event (S4 Table), 10 of whom had a concomitant documented infection. IL-6 was higher in sAH with infection than in sAH without infection (median 89.3 [IQR 46.9–381.9] vs 28.6 [15.4–37.7] pg/mL; p < 0.001) but did not differ between sAH and other ALD admissions (35.1 vs 33.5 pg/mL; p = 0.86). Only three in-hospital deaths occurred in the sAH group (2 of 10 with infection and 1 of 20 without), which precludes reliable modelling; the Firth estimate was directionally consistent with the whole cohort but imprecise (OR 1.42, 95% CI 0.72–2.68; AUC 0.679, 0.153–1.000). For 6-month (17 deaths) and overall mortality (22 deaths) in sAH, IL-6 showed no association (AUC 0.53 and 0.42; MELD-adjusted OR 0.79 [0.45–1.37] and 0.66 [0.36–1.22]). Corticosteroid exposure and Lille response were not available for these patients.

The IL-6 × ALD interaction for in-hospital mortality was unchanged after adjustment for infection and MELD-3.0 (interaction OR 1.93, 1.26–2.98; p = 0.003), after further adjustment for age and sex (1.96, 1.25–3.06; p = 0.003), and after excluding all 30 sAH patients (1.85, 1.19–2.87; p = 0.006). Within ALD, IL-6 discriminated in-hospital death in infected patients (n = 55, 20 deaths; AUC 0.741; MELD-adjusted OR 1.55, 1.00–2.39), in non-infected patients (n = 105, 6 deaths; AUC 0.685; Firth MELD-adjusted OR 1.74, 1.04–3.11) and in ALD without sAH (n = 132, 23 deaths; AUC 0.810; MELD-adjusted OR 1.75, 1.22–2.50). No IL-6 × infection interaction was detected within ALD (p = 0.90) or MASLD (p = 0.38), and no IL-6 × sAH interaction was detected (p = 0.75). In MASLD, IL-6 was not associated with in-hospital death in either infected (n = 82, 5 deaths; AUC 0.551) or non-infected patients (n = 224, 10 deaths; AUC 0.673; MELD-adjusted OR 1.19, 0.73–1.96).

In etiology-stratified mediation analysis (S5 Table), infection mediated 23.2% of the IL-6–mortality association in ALD (total OR 2.18 **→** direct OR 1.82, 1.32–2.52; infection **→** death given IL-6, OR 4.65, 1.56–13.84) but 1.6% in MASLD (total OR 1.27 **→** direct OR 1.26; infection **→** death, OR 1.04, 0.32–3.41), and 15.9% in the whole cohort after excluding sAH patients. The incremental gain from adding IL-6 to MELD-3.0 was concentrated in ALD (ΔAUC +0.053, from 0.809 to 0.862; IDI 0.103; likelihood-ratio p < 0.001), was present in ALD with and without infection (ΔAUC +0.042, p = 0.036; + 0.104, p = 0.035), and was absent in MASLD (ΔAUC +0.044 but IDI 0.001, p = 0.36), in non-infected patients (p = 0.23) and in patients with the clinical syndrome of ACLF (ΔAUC 0.000, p = 0.31).

## 5. Discussion

In this prospective observational study of 489 hospitalized patients with CLD, we found that serum IL-6 measured at admission was an independent predictor of in-hospital mortality, with consistent evidence across frequentist and Bayesian analytical frameworks. The principal findings were as follows. First, IL-6 levels exhibited a clear dose-response relationship with mortality at all time points, organ failure burden, and disease severity, with median levels approximately three-fold higher in in-hospital non-survivors compared with survivors. Second, IL-6 independently predicted in-hospital mortality after adjustment for age, sex, and MELD-3.0 though its independent contribution was attenuated for longer-term endpoints. In the fully adjusted model additionally incorporating C-reactive protein, IL-6's independent contribution was attenuated (adjusted odds ratio 1.21, p = 0.133), consistent with the substantial shared variance between IL-6 as a proximal cytokine and its downstream acute-phase effector; IL-6 retained independent significance when modelled alongside MELD-3.0 without C-reactive protein (adjusted odds ratio 1.34, p = 0.009). Third, the addition of IL-6 to MELD-3.0 yielded a statistically significant incremental improvement in discrimination for in-hospital mortality with the combined CLIF-C OF + IL-6 model achieving the highest AUC among all models tested. Fourth, a significant etiology-specific interaction was identified (interaction odds ratio 1.93, p = 0.002), with IL-6 showing substantially stronger prognostic performance in patients with ALD compared with MASLD. Fifth, Bayesian posterior analysis provided significant evidence of a positive IL-6–mortality association for in-hospital death with a temporal gradient of decreasing evidence strength for longer-term outcomes. Finally, mediation analysis indicated that infection partially mediates the IL-6–mortality relationship although the substantial residual direct effect suggests that IL-6 captures prognostic information beyond infection status alone.

The independent association between admission IL-6 and in-hospital mortality aligns with the emerging recognition that systemic inflammation is a central driver of clinical deterioration in cirrhosis, rather than hepatic dysfunction alone. IL-6 occupies a proximal position in the inflammatory cascade, being released early in response to both pathogen-associated molecular patterns from bacterial translocation and damage-associated molecular patterns from hepatocyte injury. Its elevation therefore reflects the intensity of the acute inflammatory insult at the time of hospitalization, which may explain the predictive value for short-term outcomes observed in our study.

The attenuation of IL-6’s prognostic signal for longer-term mortality endpoints (6-month, 12-month, and overall) after MELD-3.0 adjustment was an important finding that warrants mechanistic interpretation. MELD-3.0 captures the cumulative burden of hepatic and renal dysfunction, which becomes the dominant determinant of survival over time. The Bayesian temporal gradient – strong evidence for in-hospital mortality progressively weakening to moderate evidence for overall mortality suggests that IL-6 at admission functions primarily as a snapshot of the acute inflammatory milieu, whose prognostic relevance is gradually supplanted by the trajectory of organ dysfunction as captured by composite severity scores. This interpretation is further supported by the classification tree analysis, which identified MELD-3.0 as the dominant predictor with IL-6 as a complementary second feature and by the Kaplan–Meier analysis demonstrating the greatest survival separation by IL-6 tertile in the first 90 days, with subsequent convergence.

The etiology-specific interaction between IL-6 and ALD is the most novel and internally consistent finding of this study, concordant across frequentist interaction testing (p = 0.002) and Bayesian subgroup analysis. The interaction indicates that the prognostic impact of IL-6 is nearly doubled in ALD compared with other etiologies. This is biologically plausible given the distinct inflammatory phenotype of ALD, which recent syntheses of its pathogenesis attribute to coordinated cell-to-cell and organ-to-organ signalling [17]. Ethanol and its metabolite acetaldehyde disrupt intestinal tight junctions and induce dysbiosis, increasing intestinal permeability and the portal delivery of lipopolysaccharide and other microbial products; these engage Toll-like receptor 4 on Kupffer cells, which, primed by chronic alcohol exposure and by extracellular vesicles and damage-associated molecular patterns released from injured hepatocytes, amplify IL-6, tumour necrosis factor-α and CXC-chemokine production and recruit neutrophils into the liver. In parallel, alcohol-induced adipose-tissue lipolysis and inflammation increase the flux of free fatty acids and adipokines to the liver, sustaining hepatocyte stress and macrophage activation [17]. IL-6 sits at the convergence of these intrahepatic, gut–liver and adipose–liver circuits, which may explain why a single admission value carries more prognostic information in ALD than in MASLD, where the inflammatory tone is lower-grade and metabolically driven. This framework is also consistent with our etiology-stratified mediation analysis, in which infection accounted for a larger share of the IL-6–mortality association in ALD (23%) than in MASLD (2%), because bacterial translocation and clinically overt infection lie on the same gut-derived pathway. The Bayesian subgroup analysis reinforced this finding, with ALD patients demonstrating the strongest posterior evidence and MASLD patients showing the weakest. The relative attenuation in MASLD may reflect the more chronic, low-grade inflammatory profile characteristic of metabolic liver disease, in which IL-6 elevations at a single time point may be less informative about acute prognostic risk.

We examined whether the IL-6 signal is confined to, or modified by, the two precipitants most likely to raise it – bacterial infection and sAH. First, the etiology-specific effect was not an artefact of either precipitant: the IL-6 × ALD interaction was unchanged after adjustment for infection and MELD-3.0 and after excluding all sAH patients, and IL-6 discriminated in-hospital death in ALD with and without infection but not in MASLD in either stratum. Second, infection mediated 23% of the IL-6–mortality association in ALD but 2% in MASLD, where infection itself did not predict in-hospital death once IL-6 was accounted for; in ALD, admission IL-6 thus appears to integrate infection-driven and sterile (alcohol- and gut-derived) inflammation, whereas the weaker, infection-independent signal in MASLD was not clinically useful at our sample size. Third, with 30 sAH patients and three in-hospital deaths, we cannot establish whether IL-6 has special value in sAH with or without infection; the descriptive findings – IL-6 three-fold higher in sAH with infection and highest in the sAH patients who died, are compatible with the whole-cohort results but hypothesis-generating only. IL-6 showed no association with 6-month or overall mortality in sAH, where MELD-3.0 dominated, consistent with the CANONIC study finding that systemic inflammation in acute decompensation varies by precipitant and is most pronounced in alcoholic hepatitis and bacterial infection [18], and with the time-limited admission signal seen in the whole cohort. Overall, IL-6 is most informative in alcohol-associated cirrhosis irrespective of infection, of modest value in infected patients of any etiology (outperforming CRP and procalcitonin but not MELD-3.0 or CLIF-C OF), and of little value in non-infected MASLD or once organ failure is established. Whether IL-6 identifies sAH patients unlikely to respond to corticosteroids, or candidates for early transplantation, could not be addressed here and merits a dedicated study.

The mediation analysis provides insight into the mechanistic pathways linking IL-6 to mortality. The finding that infection mediated 17.3% of IL-6’s total mortality effect, while CRP mediated only 9.3%, suggests that IL-6 captures a broader prognostic signal that extends beyond either infection or downstream acute-phase inflammation alone. The substantial residual direct effect implies that IL-6 integrates information from multiple pathological processes, including sterile inflammation from hepatocyte injury, portal hypertension-driven immune activation, and bacterial translocation that may not have been clinically documented as infection. This positions IL-6 as a composite marker of the overall inflammatory burden rather than a specific indicator of any single pathological process. We also acknowledge that hepatic and extrahepatic inflammatory responses may be biologically dissociated in ACLF. Circulating IL-6 is the sum of hepatic (Kupffer-cell and hepatocyte) and extrahepatic (gut-derived, adipose, circulating monocyte and endothelial) production, and the CANONIC study analysis of systemic inflammation showed that the profile of circulating cytokines differs according to the precipitating event, being most pronounced with active alcoholism or alcoholic hepatitis and with bacterial infection, and that the severity of systemic inflammation, rather than of hepatic dysfunction alone, tracks the grade and course of ACLF [18]. A single serum measurement therefore cannot apportion the inflammatory signal to its source, and the direct effect in our mediation model should be interpreted as inflammation that is not captured by the clinical diagnosis of infection, rather than as hepatic inflammation per se; compartment-specific or tissue-level markers would be required to resolve this distinction.

Our findings substantially extend the existing literature on IL-6 as a prognostic biomarker in cirrhosis. Remmler and colleagues first demonstrated that IL-6 independently predicted 90-day and 1-year mortality in end-stage liver disease patients awaiting transplantation, reporting discriminative performance comparable to MELD [11]. Our study corroborates and extends these findings by demonstrating IL-6’s strongest predictive value for in-hospital mortality in a broader cohort that includes both transplant-eligible and non-transplant candidates across the full severity spectrum. The attenuation of IL-6’s independent predictive value for longer-term outcomes after MELD adjustment in our study aligns with the observation by Remmler et al. that IL-6 and MELD captured overlapping prognostic variance, suggesting that these measures are partly redundant for long-term prognostication.

Zhou et al. demonstrated elevated IL-6 as a mortality predictor in HBV-related ACLF, while Murakami et al. specifically identified IL-6 as prognostic in alcohol-related ACLF [15,19]. Our study extends these single-etiology observations by directly comparing the prognostic performance of IL-6 across etiologies within the same cohort and formally quantifying the interaction effect. The significantly stronger IL-6–mortality association in ALD compared with MASLD provides statistical confirmation of the etiology-specific hypothesis that has been previously suggested but not systematically tested.

The incremental discriminative improvement afforded by IL-6 over MELD-3.0 in our study was modest but statistically significant, comparable in magnitude to the increment from CRP. While such improvements may appear small in absolute terms, they are consistent with the typical magnitude of improvement reported when adding individual biomarkers to well-established prognostic models in hepatology and are clinically meaningful in the context of a single-biomarker addition to a composite score [20]. The combined CLIF-C OF + IL-6 model, which achieved an AUC of 0.802, suggests that incorporating IL-6 into organ failure–based assessment frameworks yield the most clinically useful prediction.

Our findings should also be placed in the context of recent risk-model development in hospitalized patients with cirrhosis. The LIVERAID-ICU score, derived and validated in 620 patients with cirrhosis treated for infection in an intensive care unit, combined a measure of hepatic dysfunction (Child–Pugh class) with extrahepatic physiological variables (serum urea and respiratory parameters) and achieved an AUC of 0.83 for in-hospital mortality, exceeding SOFA, MELD, Child–Pugh and CLIF-C ACLF scores [21]. Our data point in the same direction, the best-performing combination was an organ-failure-based score plus IL-6 and within infected patients IL-6 outperformed CRP and procalcitonin but not MELD-3.0 or CLIF-C OF. This suggests that the clinical niche of an IL-6-enhanced model is not to replace organ-failure-based or physiology-based scores in critically ill infected patients, where instruments such as LIVERAID-ICU already perform well, but to add an early, upstream inflammatory dimension in patients whose organ-failure burden is still limited, particularly in ALD and at intermediate MELD-3.0 scores, before extrahepatic physiological derangement becomes manifest. A head-to-head comparison with the LIVERAID-ICU score was not possible because the respiratory variables it requires were not captured in our database, and this comparison should be part of any prospective validation.

Padia et al. reported on cystatin C and IL-6 in acute decompensation, similarly, finding IL-6 associated with prognosis [22]. Our study adds to this work by employing a more comprehensive analytical framework, including mediation analysis to delineate the mechanistic pathways, Bayesian methods to provide probabilistic evidence quantification, and MELD-stratified analysis to identify the clinical scenarios in which IL-6 offers the greatest added value.

The findings of our study have several practical implications for the management of hospitalized patients with cirrhosis. First, the IL-6 threshold of 41.6 pg/mL identified here represents a candidate cutoff for admission risk stratification that requires external validation before clinical use. The lower threshold of 10 pg/mL, which achieved 90.9% sensitivity and 96.9% negative predictive value, may serve as a useful “rule-out” marker to identify patients at low risk of in-hospital death who may be suitable for less intensive monitoring. Second, the MELD-stratified analysis revealed that IL-6 provides the greatest additional prognostic information in patients with low-to-moderate MELD scores (6–24), in whom standard severity scores may underestimate the risk conferred by systemic inflammation. In contrast, among high-MELD patients (≥25), IL-6 did not significantly differentiate outcomes, likely because disease severity is already so advanced that the prognostic information is captured by MELD alone. This finding has important implications for clinical resource allocation, suggesting that IL-6 measurement may be most useful in the intermediate-severity population where clinical decision-making is most uncertain. Third, the etiology-specific interaction suggests that IL-6 should be interpreted differently in ALD versus MASLD patients. In ALD, IL-6 measurement may be particularly valuable for guiding treatment intensity and prognosis discussions. Fourth, the IL-6/albumin ratio, which marginally outperformed IL-6 alone for in-hospital mortality represents a novel composite biomarker combining inflammatory and synthetic function domains. While the improvement was incremental, this ratio has the advantage of being easily calculable from routine laboratory values and may warrant further validation as a simple bedside prognostic tool.

These results could argue against routine, unselected admission IL-6 testing. Cohort-wide gain over MELD-3.0 was small (ΔAUC +0.023) and negligible in non-infected patients (+0.003), MASLD (IDI 0.001) and established ACLF, where organ-failure scores already capture the information. In ALD cirrhosis, however, adding IL-6 to MELD-3.0 raised the AUC from 0.809 to 0.862 (IDI 0.103), and an admission value ≥41.6 pg/mL had 88.5% sensitivity, 31.1% positive and 96.5% negative predictive value for in-hospital death (positive predictive value 9.6% in MASLD). We therefore propose IL-6 as a targeted rather than routine test: of limited value in unselected patients, but warranting personalised risk-stratification research in ALD cirrhosis – particularly at intermediate MELD-3.0 (score 17–24), where standard scores are least decisive, and with documented infection, where IL-6 outperformed CRP and procalcitonin. The automated immunoassay costs about INR 1200 (USD 13) per test in our setting; formal cost-effectiveness analysis was beyond our scope, but restricting testing to ALD cirrhosis or documented infection (51% of admissions, containing 70% [31/44] of in-hospital deaths) would reduce the tests needed per additional high-risk patient identified, and assay cost must be weighed against the cost and benefit of the actions it may trigger (earlier intensive-care review, intensified infection surveillance, earlier transplant referral). Prospective studies embedding IL-6 in a management algorithm with economic evaluation are needed before any clinical recommendation.

## 6. Limitations and future directions

Several limitations should be acknowledged. First, this was a single-center observational study conducted at a tertiary hepatology referral center, which may limit the generalizability of our findings to community hospitals or primary care settings where disease severity and etiology distributions may differ. The predominance of MASLD and ALD in our cohort reflected the evolving epidemiology of liver disease in our region but means that findings may not be directly applicable to populations with predominantly viral hepatitis etiologies. Second, IL-6 was measured only at admission, and serial measurements were not available. The single time-point design precludes assessment of whether dynamic changes in IL-6 over the hospital course provide additional prognostic information beyond the admission value, which is a limitation given the known fluctuating nature of IL-6 in acute illness. Third, the overall mortality rate in our cohort was high, reflecting the tertiary referral nature and advanced disease severity of the population. This may limit the applicability of our identified cutoffs in populations with lower baseline mortality. Fourth, while we adjusted for multiple confounders, residual confounding from unmeasured variables, such as specific antimicrobial therapies, vasoactive medications, or nutritional status, cannot be entirely excluded. In addition, corticosteroid exposure and day-7 Lille response were not recorded for patients with sAH, so we cannot report how many were admitted as corticosteroid non-responders or examine whether IL-6 relates to steroid responsiveness. Fifth, the relatively modest incremental AUC improvement from IL-6 raises questions about cost-effectiveness in routine clinical practice. Because the reclassification benefit was confined to identifiable subgroups, a cost-effectiveness argument for routine measurement cannot be made from these data; the sub-cohort analyses were post hoc, involved small numbers of events in several strata, were not corrected for multiplicity, and require prospective confirmation in etiology- and infection-enriched cohorts. However, the significant likelihood ratio test improvement and the IDI suggest that the reclassification benefit may be clinically meaningful even when the AUC change appears small. Sixth, we did not have access to other emerging biomarkers for direct comparison, which would have provided additional context for the relative utility of IL-6 in a multi-biomarker panel. Finally, external validation in independent cohorts with different demographic and etiological profiles is necessary before these results can be translated into clinical practice guidelines.

More broadly, our findings should be read in the light of the methodological limitations that characterise prognostic-biomarker research in cirrhosis. A systematic review evaluating candidate biomarkers found substantial heterogeneity in study design, outcome definitions and analytical methods, frequent reliance on single-centre derivation cohorts and data-driven thresholds, and a lack of external validation, and called for standardised evaluation frameworks [23]. Although that review addressed compensated cirrhosis, its conclusions apply directly to the present study: the IL-6 cut-offs (41.6 and 10 pg/mL) and the IL-6/albumin ratio proposed here were derived post hoc in a single cohort, are optimistic by construction, are assay-dependent, and must be regarded as candidate rather than validated tools until they have been tested prospectively in independent cohorts with prespecified thresholds, harmonised outcome definitions, and assessment of calibration and clinical utility. A systematic review and meta-analysis of biomarkers in decompensated cirrhosis reached similar methodological conclusions while identifying IL-6 as the biomarker with the largest ratio of means between 90-day non-survivors and survivors [24], which supports IL-6 as a reasonable candidate for such validation but does not substitute for it.

Several specific avenues for future investigation emerge from this work. First, serial IL-6 measurement studies are needed to determine whether temporal trends in IL-6 (e.g., change from admission to 48–72 hours) provide superior prognostic information compared with single baseline measurements. Second, prospective studies specifically enriched for ALD and sAH patients, with systematic capture of corticosteroid exposure and day-7 Lille response, should validate the etiology-specific prognostic performance observed here. Third, multi-biomarker panels incorporating IL-6 alongside complementary markers such as cystatin C, presepsin, and markers of intestinal barrier dysfunction could be evaluated to determine whether a composite inflammatory–organ dysfunction score can substantially outperform existing models. Fourth, interventional studies examining whether IL-6 directed therapeutic strategies, such as targeted anti-inflammatory therapy or early ICU escalation in patients with markedly elevated IL-6, can modify outcomes are warranted. Fifth, the IL-6/albumin ratio requires external validation across diverse populations and comparison against other composite indices to establish its clinical utility.

## 7. Conclusion

In this study of 489 hospitalized patients with cirrhosis, serum interleukin-6 at admission was an independent predictor of in-hospital mortality, supported by both frequentist and Bayesian evidence. MELD-3.0 remained the dominant prognostic determinant, and interleukin-6 did not outperform established severity scores in isolation. The addition of interleukin-6 to established severity scores modestly but significantly improved short-term mortality discrimination, with the combined Chronic Liver Failure Consortium Organ Failure score plus interleukin-6 model achieving the highest predictive performance among all models tested. A significant etiology-specific interaction was identified, with interleukin-6 showing nearly double the prognostic impact in ALD compared with MASLD, highlighting the role of the underlying inflammatory phenotype in modifying biomarker utility. These findings position interleukin-6 as a candidate complement to, not a substitute for, established severity scores for short-term risk assessment. Routine measurement in unselected admissions is not supported by these data; targeted use in alcohol-associated cirrhosis – particularly with documented infection or intermediate MELD-3.0 scores – is the setting in which interleukin-6 merits prospective validation with embedded economic evaluation before clinical adoption.

## Supporting information

S1 Fig Median serum IL-6 levels at admission stratified by the presence or absence of clinical features. Features shown: infection, mechanical ventilation, hypotension, acute kidney injury (AKI), hepatic encephalopathy (HE), medical intensive care unit (MICU) admission, refractory ascites, and ascites. All comparisons p < 0.001, Mann–Whitney U test.
(TIF)

S2 Fig Relationship between IL-6 and organ failure burden at admission. Median IL-6 values (with interquartile range) are plotted against the number of organ failures (0–6). Sample sizes are annotated at each point. Spearman rank correlation coefficient ρ = 0.390, p < 0.001.
(TIF)

S3 Fig Sensitivity and specificity of serum IL-6 at selected cutoff values for predicting in-hospital mortality. The Youden-optimal cutoff was 41.6 pg/mL (sensitivity 77%, specificity 66%). Lower cutoffs (≥10, ≥ 20 pg/mL) favored sensitivity; higher cutoffs (≥50, ≥ 100 pg/mL) favored specificity.
(TIF)

S4 Fig Median serum IL-6 levels by in-hospital survival status and disease etiology. In alcohol-associated liver disease (ALD; n = 160), non-survivors had a median IL-6 of 127 pg/mL compared with 27 pg/mL in survivors. In metabolic dysfunction-associated steatotic liver disease (MASLD; n = 306), the corresponding values were 54 and 20 pg/mL. The IL-6 × ALD interaction was significant (OR = 1.93, p = 0.002), indicating a stronger IL-6–mortality association in ALD than in MASLD.
(TIF)

S1 Table Admission pathway, presenting problems, precipitants, level of care and special populations in the study cohort (N = 489). Presenting complaints and precipitants are not mutually exclusive. Precipitants are those recorded by the treating team at admission; the absence of a recorded precipitant does not exclude one. ICU, intensive care unit; HCC, hepatocellular carcinoma; sAH, severe alcohol-associated hepatitis.
(DOCX)

S2 Table Infections documented at admission: site, organism, admission IL-6 and in-hospital mortality (n = 145). Site categories were assigned from the recorded site of infection; 21 patients had infection at two or more sites, so percentages sum to more than 100. Organism counts include polymicrobial isolates (each organism counted once). IL-6 in pg/mL, median (IQR). SARS-CoV-2 infections reflect the overlap of the study period with the COVID-19 pandemic.
(DOCX)

S3 Table Incremental value of IL-6 over MELD-3.0 and cut-off (41.6 pg/mL) performance by stratum. ΔAUC = AUC(MELD-3.0 + log IL-6) − AUC(MELD-3.0). IDI, integrated discrimination improvement. LR test, likelihood-ratio test for the addition of log IL-6 to MELD-3.0. Sensitivity, specificity, positive (PPV) and negative (NPV) predictive values for the Youden-optimal whole-cohort cut-off of 41.6 pg/mL applied within each stratum.
(DOCX)

S4 Table Characteristics and outcomes of sAH vs other ALD vs whole cohort. sAH vs. ALD without sAH: age p < 0.001; bilirubin p = 0.003; MELD-3.0 p = 0.030; IL-6 p = 0.86 (Mann–Whitney U); in-hospital mortality p = 0.42 (Fisher's exact test). All 30 had ACLF at admission. Ten sAH patients had a concomitant documented infection (IL-6 89.3 [46.9–381.9] vs 28.6 [15.4–37.7] pg/mL without infection; p < 0.001). Corticosteroid exposure and Lille response were not recorded.
(DOCX)

S5 Table Baron–Kenny mediation by infection, stratified by etiology. Path c: log IL-6 → in-hospital death (unadjusted). Path a: log IL-6 → documented infection at admission. Path b: infection → in-hospital death, adjusted for log IL-6. Path c′: log IL-6 → in-hospital death, adjusted for infection. Proportion mediated = (c − c′)/c on the log-odds scale. Within infected patients (n = 144 with CRP available, 25 deaths), log CRP as mediator accounted for 5.3% of the IL-6 effect (total OR 1.53 → direct OR 1.50, 95% CI 1.14–1.98; p = 0.004).
(DOCX)
