# Supplementary material for: Admission interleukin-6 as a time-limited, etiology-dependent prognostic signal in hospitalized cirrhosis – A frequentist and Bayesian cohort study
Source: PLoS One. 2026 Oct 8;21(10):e0360005. doi: 10.1371/journal.pone.0360005 (PMC13648997; doi:10.1371/journal.pone.0360005)
Supplement: Supplementary file 1 [file pone.0360005.s005.docx]

**Supplementary Table 1:** Admission pathway, presenting problems, precipitants, level of care and special populations in the study cohort

| **Settings and patient pathways** | **N (%)** |
| --- | --- |
| Route of admission |  |
| Emergency/unplanned admission | 341 (69.7%) |
| Planned admission via outpatient clinic | 148 (30.3%) |
| Presenting complaint(s) prompting admission (not mutually exclusive) |  |
| Ascites | 148 (30.3%) |
| Suspected/confirmed infection or sepsis | 112 (22.9%) |
| Variceal bleeding | 90 (18.4%) |
| Jaundice | 74 (15.1%) |
| Hepatic encephalopathy | 73 (14.9%) |
| HCC-related | 33 (6.7%) |
| Acute kidney injury | 6 (1.2%) |
| Hyponatraemia | 4 (0.8%) |
| Hypotension | 4 (0.8%) |
| Other/multifactorial | 13 (2.7%) |
| Identifiable acute precipitant recorded | 110 (22.5%) |
| Bacterial infection / septic shock | 43 (8.8%) |
| Severe alcohol-associated hepatitis (sAH) | 30 (6.1%) |
| Complementary and alternative medicine (CAM)-related hepatotoxicity | 26 (5.3%) |
| Drug-induced liver injury (conventional drugs) | 6 (1.2%) |
| Viral hepatitis reactivation/flare (HBV/HCV/acute viral hepatitis) | 7 (1.4%) |
| Other (hypotension, surgery) | 3 (0.6%) |
| Clinical syndrome at admission |  |
| Compensated cirrhosis | 39 (8.0%) |
| Decompensated cirrhosis | 338 (69.1%) |
| Acute-on-chronic liver failure | 112 (22.9%) |
| Level of care and organ support |  |
| Direct admission to medical ICU | 150 (30.7%) |
| Transfer to ICU during hospital stay | 151 (30.9%) |
| Hypotension at admission | 31 (6.3%) |
| Mechanical ventilation at admission / during stay | 15 (3.1%) / 33 (6.7%) |
| Renal replacement therapy at admission / during stay | 9 (1.8%) / 16 (3.3%) |
| Acute variceal bleeding at admission | 91 (18.6%) |
| Documented infection at admission | 145 (29.7%) |
| Special populations |  |
| Hepatocellular carcinoma | 97 (19.8%) |
| Transition to do-not-resuscitate status during admission | 26 (5.3%) |
| Outcomes |  |
| In-hospital mortality | 44 (9.0%) |
| Length of stay among survivors, median (IQR) days | 5 (3–7) |
