# Supplementary material for: Admission interleukin-6 as a time-limited, etiology-dependent prognostic signal in hospitalized cirrhosis – A frequentist and Bayesian cohort study
Source: PLoS One. 2026 Oct 8;21(10):e0360005. doi: 10.1371/journal.pone.0360005 (PMC13648997; doi:10.1371/journal.pone.0360005)
Supplement: Supplementary file 2 [file pone.0360005.s006.docx]

**Supplementary Table 2:** Infections documented at admission: site, organism, IL-6 and in-hospital mortality

| **Characteristics** | **Value** |
| --- | --- |
| Patients with documented infection at admission | 145 (29.7% of 489) |
| Site of infection (not mutually exclusive; 21 patients had ≥2 sites) |  |
| Bloodstream infection (bacteraemia/fungaemia) | 76 (52.4%) |
| Urinary tract infection | 51 (35.2%) |
| Respiratory tract infection (pneumonia/LRTI, pleural) | 13 (9.0%) |
| Spontaneous bacterial peritonitis | 12 (8.3%) |
| Skin and soft-tissue infection | 12 (8.3%) |
| Bone and joint infection | 3 (2.1%) |
| Infective gastroenteritis | 1 (0.7%) |
| Organism identified (any site) | 99 (68.3%) |
| Klebsiella pneumoniae | 25 |
| Candida spp./other fungi | 18 |
| Escherichia coli | 15 |
| Enterococcus spp. | 5 |
| SARS-CoV-2 | 5 |
| Enterobacter spp. | 4 |
| Staphylococcus spp. | 3 |
| Leptospira spp. | 2 |
| Streptococcus spp. | 2 |
| Pseudomonas aeruginosa | 2 |
| Salmonella spp. | 2 |
| Acinetobacter baumannii | 1 |
| IL-6 (pg/mL), median (IQR): infected vs non-infected | 63.1 (21.6–199.8) vs 17.0 (6.8–46.1); p < 0.001 |
| In-hospital mortality: infected vs non-infected | 25/145 (17.2%) vs 19/344 (5.5%) |
| IL-6 median (IQR) — Bloodstream infection | 91.0 (32.0–323.6) [n=76; in-hospital deaths 17] |
| IL-6 median (IQR) — Urinary tract infection | 46.1 (20.8–90.3) [n=51; in-hospital deaths 3] |
| IL-6 median (IQR) — Respiratory tract infection | 74.6 (45.6–206.5) [n=13; in-hospital deaths 3] |
| IL-6 median (IQR) — Spontaneous bacterial peritonitis | 203.8 (59.6–827.0) [n=12; in-hospital deaths 1] |
| IL-6 median (IQR) — Skin and soft-tissue infection | 39.5 (11.2–146.5) [n=12; in-hospital deaths 2] |
