# Supplementary material for: Admission interleukin-6 as a time-limited, etiology-dependent prognostic signal in hospitalized cirrhosis – A frequentist and Bayesian cohort study
Source: PLoS One. 2026 Oct 8;21(10):e0360005. doi: 10.1371/journal.pone.0360005 (PMC13648997; doi:10.1371/journal.pone.0360005)
Supplement: Supplementary file 3 [file pone.0360005.s007.docx]

**Supplementary Table 3:** Incremental value of IL-6 over MELD-3.0 and cut-off (41.6 pg/mL) performance by stratum

| **Stratum** | **n (deaths)** | **AUC MELD-3.0 alone** | **AUC MELD-3.0 + IL-6** | **ΔAUC** | **IDI** | **LR-test p** | **IL-6 ≥41.6 pg/mL: sensitivity %** | **Specificity %** | **PPV %** | **NPV %** |
| --- | --- | --- | --- | --- | --- | --- | --- | --- | --- | --- |
| Whole cohort | 489 (44) | 0.762 | 0.785 | +0.023 | 0.0221 | 0.008 | 77.3 | 66.1 | 18.4 | 96.7 |
| Documented infection at admission | 145 (25) | 0.732 | 0.763 | +0.032 | 0.0203 | 0.071 | 92.0 | 42.5 | 25.0 | 96.2 |
| No infection at admission | 344 (19) | 0.746 | 0.749 | +0.003 | 0.0054 | 0.232 | 57.9 | 74.8 | 11.8 | 96.8 |
| ALD (all) | 160 (26) | 0.809 | 0.862 | +0.053 | 0.1031 | <0.001 | 88.5 | 61.9 | 31.1 | 96.5 |
| ALD with infection | 55 (20) | 0.812 | 0.854 | +0.042 | 0.0568 | 0.036 | 95.0 | 40.0 | 47.5 | 93.3 |
| ALD without infection | 105 (6) | 0.604 | 0.707 | +0.104 | 0.0910 | 0.035 | 66.7 | 69.7 | 11.8 | 97.2 |
| MASLD (all) | 306 (15) | 0.623 | 0.667 | +0.044 | 0.0010 | 0.356 | 66.7 | 67.7 | 9.6 | 97.5 |
| MASLD with infection | 82 (5) | 0.757 | 0.758 | +0.001 | 0.0046 | 0.463 | 80.0 | 42.9 | 8.3 | 97.1 |
| MASLD without infection | 224 (10) | 0.765 | 0.762 | -0.003 | 0.0026 | 0.496 | 60.0 | 76.6 | 10.7 | 97.6 |
| ACLF (clinical syndrome) | 112 (16) | 0.788 | 0.788 | +0.000 | 0.0148 | 0.308 | 81.2 | 58.3 | 24.5 | 94.9 |
| sAH (all) | 30 (3) | 0.815 | 0.840 | +0.025 | -0.0024 | 0.535 | 66.7 | 59.3 | 15.4 | 94.1 |
