# Supplementary material for: Admission interleukin-6 as a time-limited, etiology-dependent prognostic signal in hospitalized cirrhosis – A frequentist and Bayesian cohort study
Source: PLoS One. 2026 Oct 8;21(10):e0360005. doi: 10.1371/journal.pone.0360005 (PMC13648997; doi:10.1371/journal.pone.0360005)
Supplement: Supplementary file 4 [file pone.0360005.s008.docx]

**Supplementary Table 4:** Characteristics and outcomes of sAH vs other ALD vs whole cohort

| **Characteristic** | **sAH (n=30)** | **ALD without sAH (n=132)** | **Whole cohort (N=489)** |
| --- | --- | --- | --- |
| Age, years | 48 (42–54) | 59 (51–66) | 62 (54–68) |
| Male sex | 30 (100.0%) | 130 (98.5%) | 418 (85.5%) |
| Total bilirubin, mg/dL | 10.9 (4.4–19.9) | 5.2 (2.9–10.4) | 3.3 (1.6–6.6) |
| AST, IU/L | 104 (64–150) | 86 (52–137) | 66 (45–115) |
| INR | 2.06 (1.71–2.63) | 1.94 (1.55–2.42) | 1.71 (1.42–2.24) |
| Creatinine, mg/dL | 1.2 (0.9–1.6) | 1.1 (0.8–1.8) | 1.1 (0.8–1.7) |
| MELD-3.0 | 26 (24–32) | 22 (18–28) | 20 (15–26) |
| CTP score | 11 (10–12) | 10 (9–12) | 10 (8–11) |
| CLIF-C OF score | 8 (7–10) | 8 (6–9) | 7 (6–9) |
| Number of organ failures at admission | 2 (2–3) | 2 (1–2) | 2 (1–2) |
| CRP, mg/L | 21.1 (8.8–50.9) | 33.4 (9.6–58.8) | 18.1 (5.8–44.7) |
| Procalcitonin, ng/mL | 0.50 (0.18–1.00) | 0.50 (0.20–1.90) | 0.30 (0.10–0.90) |
| IL-6, pg/mL | 35.1 (18.8–65.5) | 33.5 (12.2–108.3) | 23.8 (9.4–71.8) |
| Documented infection at admission | 10 (33.3%) | 47 (35.6%) | 145 (29.7%) |
| Hepatic encephalopathy at admission | 11 (36.7%) | 39 (29.5%) | 131 (26.8%) |
| Acute kidney injury at admission | 7 (23.3%) | 40 (30.3%) | 103 (21.1%) |
| Direct MICU admission | 9 (30.0%) | 54 (40.9%) | 150 (30.7%) |
| In-hospital mortality | 3 (10.0%) | 23 (17.4%) | 44 (9.0%) |
| 6-month mortality | 17 (56.7%) | 93 (70.5%) | 295 (60.3%) |
| Overall mortality during follow-up | 22 (73.3%) | 98 (74.2%) | 345 (70.6%) |
