# Supplementary material for: Admission interleukin-6 as a time-limited, etiology-dependent prognostic signal in hospitalized cirrhosis – A frequentist and Bayesian cohort study
Source: PLoS One. 2026 Oct 8;21(10):e0360005. doi: 10.1371/journal.pone.0360005 (PMC13648997; doi:10.1371/journal.pone.0360005)
Supplement: Supplementary file 5 [file pone.0360005.s009.docx]

**Supplementary Table 5:** Baron–Kenny mediation by infection, stratified by etiology

| **Stratum** | **n (deaths)** | **Path c: total effect, OR (95% CI)** | **Path a: IL-6 → infection, OR (95% CI)** | **Path b: infection → death given IL-6, OR (95% CI)** | **Path c′: direct effect, OR (95% CI)** | **Proportion mediated by infection** |
| --- | --- | --- | --- | --- | --- | --- |
| Whole cohort | 489 (44) | 1.61 (1.34–1.93) | 1.69 (1.47–1.95) | 2.08 (1.04–4.19); p = 0.040 | 1.48 (1.22–1.80); p = <0.001 | 17.4% |
| ALD | 160 (26) | 2.18 (1.58–3.00) | 1.83 (1.42–2.35) | 4.65 (1.56–13.84); p = 0.006 | 1.82 (1.32–2.52); p = <0.001 | 23.2% |
| MASLD | 306 (15) | 1.27 (0.94–1.72) | 1.59 (1.33–1.89) | 1.04 (0.32–3.41); p = 0.949 | 1.26 (0.91–1.75); p = 0.156 | 1.6% |
| Whole cohort excluding sAH | 459 (41) | 1.62 (1.34–1.96) | 1.65 (1.43–1.89) | 2.04 (1.00–4.19); p = 0.051 | 1.50 (1.23–1.84); p = <0.001 | 15.9% |
