# Supplementary material for: Admission interleukin-6 as a time-limited, etiology-dependent prognostic signal in hospitalized cirrhosis – A frequentist and Bayesian cohort study
Source: PLoS One. 2026 Oct 8;21(10):e0360005. doi: 10.1371/journal.pone.0360005 (PMC13648997; doi:10.1371/journal.pone.0360005)
Supplement: Supplementary file 6 [file pone.0360005.s010.docx]

**RESPONSE TO REVIEWERS**

Dear Editor,

We thank the Editor and Reviewer #1 for a careful and constructive review. The comments have led us to (i) report the study explicitly against the STROBE statement; (ii) restructure the Patients section so that eligibility criteria, the clinical setting and the usual admission pathways are presented first; (iii) add explicit definitions for severe alcohol-associated hepatitis (sAH) and for the five most frequent infections in the cohort, together with the departmental standard of care; (iv) analyse the infection, sAH and sAH-plus-infection sub-cohorts separately, including etiology-stratified mediation and interaction-robustness analyses, all computed from the original study database; (v) move interpretive sentences out of the Results; and (vi) add the requested Discussion material on the “broken” cohort, on personalised versus routine IL-6 use, on cost-effectiveness, and on the five literature-based points raised by the Editor.

Every comment is reproduced below, followed by our response and by the exact change made to the manuscript, with its location. Where an item requested by the reviewer was not captured in the study database, we say so rather than reconstruct it retrospectively, and we have added the corresponding limitation.

We hope that the revised manuscript is now suitable for publication in PLOS ONE.

# **Response to Reviewer #1**

## **Comment 1**

**Comment:** *Your paper is implicitly well-aligned with the STROBE Extension, please mention it explicitly and in all the domains where appropriate.*

**Response**

We agree. The revised manuscript now states explicitly that the study is reported in accordance with the STROBE statement for cohort studies. Beyond the explicit statement, we have strengthened the STROBE domains that were only implicit in the original submission.

**Changes made**

**(a) Section 2.1,** first paragraph after the sentence ending “…consecutive design was adopted to minimize selection bias.” – added:

The study is reported in accordance with the Strengthening the Reporting of Observational Studies in Epidemiology (STROBE) statement for cohort studies; the completed checklist is provided as Supplementary Document 1.

**(b1) Section 3.1 (Basic Statistics)**, end of paragraph, added:

No formal a priori sample-size calculation was performed; the sample comprised all consecutive eligible admissions during the study period.

**(b2) Section 4.1,** end of paragraph, added:

The primary adjusted model (log IL-6, age, sex and MELD-3.0) had approximately 11	events per variable. C-reactive protein was missing in 21 patients (4.3%) and procalcitonin	in 52 (10.6%); analyses involving these variables were performed on complete cases, and	the denominators are reported with each estimate. All other variables were complete.

**(c) Section 4.1, first sentence**, replaced “A total of 489 consecutive patients hospitalized with cirrhosis were included in this study.” with:

During the study period, 828 consecutive admissions of patients with suspected or known liver disease were screened; 339 were excluded (age <18 years, N=23; no liver disease, N=66; non-cirrhotic liver disease, N=183; IL-6 not measured at admission, N=56; prior liver transplantation, N=11), leaving 489 patients for analysis.

## **Comment 2**

**Comment:** *2.1. Patients. Provide phenotypes of patients with the following points in mind: start with incl/exclusion criteria; already at this stage of reading, readers should be oriented what is the usual flow of your (considerable) enrolees per time: patient pathways/reasons for admission; proportion of acute in-comers from your catchment area (what is it) and what are the usual acute complications you are asked to manage; what proportion of patients is admitted for the second-line treatments from the secondary in-hospital care (e.g., failure to control bleeding, resistant infections, extracorporeal modalities; preemptive/rescue TIPS, etc); liver transplant decision-making (do you provide LT, and if yes, early LT for sAH?); HCC management; etc.; second opinion; deciding futility, palliative care, EOLT (you mention in passing DNR in 4.12.).*

**Response**

We agree. A new Table 1, that quantifies, from the study database, the route of admission, the presenting complaints, the identifiable acute precipitants, the level of care and organ support at admission and during the stay, the proportion with hepatocellular carcinoma, and the proportion transitioning to do-not-resuscitate status. Where specific patient data seemed overstated or redundant, not imperative to the paper’s objectives, or was not captured, it was not included.

**Changes made**

**Section 2.1:** Inclusion criteria were: (1) age ≥18 years; (2) a diagnosis of cirrhosis as defined in Section 2.2; (3) hospitalisation at the study centre between April 2022 and October 2023; and (4) measurement of serum IL-6 within 24 hours of admission as part of the standard admission laboratory panel. Exclusion criteria were (1) absence of liver disease and non-cirrhotic portal hypertension; (2) prior liver transplantation; (3) IL-6 not measured as per protocol; (4) patients admitted for elective procedures and evaluations were eligible provided they met the criteria.

**Section 4.1**: A new Table 1 has been added – “Admission pathway, presenting problems, precipitants, level of care and special populations in the study cohort”.

## **Comment 3**

**Comment:** *sAH: I suppose, you have patients with severe alcohol-associated hepatitis: provide definition you use, and (in results and discussion) how many of them you´ve had, how many were admitted as a non-responders to CS.*

**Response**

Severe alcohol-associated hepatitis was the recorded acute precipitant in 30 patients (6.1% of the cohort; 28 of the 160 patients with ALD as the underlying etiology [17.5%]. All 30 fulfilled the criteria for ACLF at admission. Their characteristics are now given in a new Supplementary Table 1 and summarised in the new Results section 4.1.

Regarding corticosteroid non-responders: our study database recorded the precipitant and the admission IL-6 value, but did not capture corticosteroid exposure before admission or the day-7 Lille response, because IL-6 was measured at admission before any treatment decision and the study was designed to evaluate admission IL-6 rather than treatment response. We therefore cannot state how many sAH patients were admitted as corticosteroid non-responders, and we now say so explicitly in the Results and in the Limitations rather than reconstruct this retrospectively.

**Changes made**

**(a) Section 2.2**, inserted:

Severe alcohol-associated hepatitis (sAH) was defined according to the National Institute on Alcohol Abuse and Alcoholism (NIAAA) Alcoholic Hepatitis Consortia criteria and the 2019 AASLD practice guidance. Severe AH was diagnosed in patients with a Maddrey discriminant function ≥32.

**(b) Section 4.1**, inserted:

An identifiable acute precipitant was recorded in 110 patients (22.5%): bacterial infection or septic shock in 43 (8.8%), sAH in 30 (6.1%), hepatotoxicity from complementary and alternative medicines in 26 (5.3%), conventional drug-induced liver injury in 6 (1.2%) and viral hepatitis flare or reactivation in 7 (1.4%). All 30 patients with sAH had ACLF at admission. The admission pathway, presenting problems, precipitants, level of care and special populations in the study cohort (Supplementary Table 1).

**(c) Section 6 (Limitations)**, inserted:

In addition, corticosteroid exposure and day-7 Lille response were not recorded for patients with sAH, so we cannot report how many were admitted as corticosteroid non-responders or examine whether IL-6 relates to steroid responsiveness.

**(d) Section 6 (Future Directions)**, replaced “Second, prospective studies specifically enriched for ALD patients should validate the etiology-specific prognostic performance observed here” with:

Second, prospective studies specifically enriched for ALD and sAH patients, with systematic capture of corticosteroid exposure and day-7 Lille response, should validate the etiology-specific prognostic performance observed here

## **Comment 4**

**Comment:** *infections: provide explicit definitions and SOC of at least 5 most common infections.*

**Response**

We have replaced the single sentence on infection with explicit, site-specific diagnostic definitions and a description of our departmental standard of care for the most frequent infections in the cohort.

**Changes made**

**Section 2.2** replaced the sentence “Infection was diagnosed based on positive cultures, radiological evidence, or clinical and laboratory criteria consistent with established site-specific definitions for spontaneous bacterial peritonitis, urinary tract infection, pneumonia, bacteraemia, and skin and soft tissue infections.” with:

Infection at admission was defined as a clinically, microbiologically and/or radiologically documented infection present at, or diagnosed within 48 hours of, admission, using the site-specific criteria – Bloodstream infection: growth of a recognised pathogen in at least one blood culture, or of a common skin commensal in at least two separately drawn cultures, with compatible clinical features; Urinary tract infection: urinary or systemic symptoms with >10 leukocytes per high-power field on urinalysis and a positive urine culture (≥10⁵ colony-forming units/mL), or, in catheterised patients, a positive culture with pyuria; pneumonia and lower respiratory tract infection: a new or progressive pulmonary infiltrate on chest imaging plus at least two of fever or hypothermia, leukocytosis or leukopenia, purulent sputum and worsening oxygenation; spontaneous bacterial peritonitis: an ascitic-fluid polymorphonuclear count ≥250 cells/mm³ in the absence of a surgically treatable intra-abdominal source, irrespective of the culture result; and skin and soft-tissue infection: clinical cellulitis, erysipelas or necrotising infection (localised erythema, warmth, tenderness and swelling with or without fever), with or without a positive wound or tissue culture. Blood, urine and (when ascites was present) ascitic-fluid cultures and chest imaging were obtained before antibiotics in all patients with suspected infection. Empirical therapy followed the departmental protocol, aligned with the standard regional guidance, adapted to local antimicrobial resistance patterns: for community-acquired spontaneous bacterial peritonitis, urinary tract infection and pneumonia, a third-generation cephalosporin (ceftriaxone), with a macrolide added for pneumonia; for healthcare-associated or nosocomial infections and for septic shock, piperacillin–tazobactam or a carbapenem (meropenem), with a glycopeptide (vancomycin or teicoplanin) when methicillin-resistant Staphylococcus aureus or enterococcal infection was suspected, and an echinocandin in septic shock with risk factors for invasive candidiasis; de-escalation to pathogen-directed therapy within 48–72 hours according to culture results; for spontaneous bacterial peritonitis, intravenous albumin 1.5 g/kg on day 1 and 1 g/kg on day 3 in addition to antibiotics, followed by secondary prophylaxis with norfloxacin; for skin and soft-tissue infection, a cephalosporin or amoxicillin–clavulanate, with clindamycin or vancomycin for suspected MRSA and urgent surgical debridement for necrotising infection; for bloodstream infection, pathogen-directed therapy for 10–14 days with removal of intravascular devices; for urinary tract infection, a third-generation cephalosporin or a fluoroquinolone for 5–7 days according to susceptibility; for pneumonia, 7 days of therapy with review of oxygen requirement and ventilatory support.

**Section 4.4**, end of the first paragraph, inserted:

Site categories in this paragraph are mutually exclusive, as recorded at admission…A harmonised any-site tabulation, in which infections at more than one site contribute to each category, together with site-specific IL-6 levels and in-hospital mortality, is provided in Supplementary Table 2.

## **Comment 5**

**Comment:** *Both these sub-cohorts analyze separately and, also sAH+infection as a trigger for admission: this might be another very important result as to the individual/personalized use of IL-6 with a different mediation analysis results.*

**Response**

We thank the reviewer for this suggestion, which we have implemented in full using the original patient-level dataset. A new Results section (4.16, “IL-6 in clinically enriched sub-cohorts”) and five new Supplementary Tables (S4–S8) report IL-6 separately in (i) patients with documented infection at admission (n = 145), (ii) patients without infection (n = 344), (iii) patients with sAH (n = 30), (iv) sAH with (n = 10) and without (n = 20) infection, (v) ALD with and without infection and ALD without sAH, (vi) MASLD with and without infection, and (vii) the clinical syndrome of ACLF with and without infection. For each stratum we give the number of deaths, IL-6 in survivors and non-survivors, the AUC with DeLong 95% confidence interval, the unadjusted and MELD-3.0-adjusted odds ratio per log-unit of IL-6, the incremental value of IL-6 over MELD-3.0 (ΔAUC, IDI, likelihood-ratio test), the performance of the cohort-derived cut-off of 41.6 pg/mL, and — for the mediation question — the Baron–Kenny decomposition of the IL-6–mortality association through infection, repeated within ALD and MASLD, and the IL-6 × ALD interaction re-estimated after adjustment for infection and MELD-3.0 and after excluding all sAH patients. Because several strata contain fewer than ten deaths, we used Firth's penalised-likelihood logistic regression with profile-likelihood confidence intervals in those strata (marked † in the tables) and did not attempt MELD-adjusted models where fewer than five deaths were available; these analyses were post hoc and are presented as hypothesis-generating.

In short, the etiology-specific signal is not an artefact of sAH or of infection; IL-6 integrates both infection-driven and sterile inflammation in ALD, whereas in MASLD the weak signal is infection-independent and not clinically useful at our sample size; and the sAH subgroup is too small for a conclusion about “special value”, although the descriptive data are compatible with the whole-cohort findings. These results are the basis of the new Discussion paragraphs requested in Comments 7–9.

**Changes made**

**(a) Section 3.2 (Advanced Statistics)**, end of the first paragraph, inserted:

Post hoc sub-cohort analyses requested at peer review examined IL-6 separately in patients with documented infection at admission, in patients with sAH, in the combination of the two, and within etiology strata, using the same logistic-regression and ROC methods; the Baron and Kenny mediation analysis and the IL-6 × etiology interaction were repeated within strata and after adjustment for infection and MELD-3.0. Where a stratum contained fewer than 10 outcome events, Firth's bias-reduced penalised-likelihood logistic regression with profile-likelihood confidence intervals was used, and MELD-adjusted estimates were not attempted with fewer than five events. These analyses were exploratory, were not corrected for multiplicity, and are interpreted as hypothesis-generating.

**(b) New Section 4.16**, inserted after Section 4.15 and before “5. DISCUSSION”:

**4.16 IL-6 in clinically enriched sub-cohorts: infection, severe alcohol-associated hepatitis and etiology**

IL-6 was examined separately in patients with documented infection at admission, in patients with sAH, in their combination, and within etiology strata. Among the 145 patients with infection (25 in-hospital deaths, 17.2%), IL-6 discriminated in-hospital death (AUC 0.700, 95% CI 0.595–0.805; unadjusted OR per log-unit 1.54, 1.18–2.00), with attenuation after MELD-3.0 adjustment (OR 1.30, 0.97–1.74; p = 0.075). In this stratum the AUC of IL-6 exceeded that of CRP (0.606) and procalcitonin (0.612) but not that of MELD-3.0 (0.732) or CLIF-C OF (0.749). Among the 344 non-infected patients (19 deaths, 5.5%), discrimination was weaker (AUC 0.627, 0.490–0.763; MELD-adjusted OR 1.24, 0.88–1.75). Adding IL-6 to MELD-3.0 increased the AUC by +0.032 in infected patients (likelihood-ratio test p = 0.071) and by +0.003 in non-infected patients (p = 0.23). The cut-off of 41.6 pg/mL had a sensitivity of 92.0% and a negative predictive value of 96.2% in infected patients, but a sensitivity of 57.9% in non-infected patients (Supplementary Table 3).

Thirty patients had sAH as the precipitating event (Supplementary Table 4), 10 of whom had a concomitant documented infection. IL-6 was higher in sAH with infection than in sAH without infection (median 89.3 [IQR 46.9–381.9] vs 28.6 [15.4–37.7] pg/mL; p < 0.001) but did not differ between sAH and other ALD admissions (35.1 vs 33.5 pg/mL; p = 0.86). Only three in-hospital deaths occurred in the sAH group (2 of 10 with infection and 1 of 20 without), which precludes reliable modelling; the Firth estimate was directionally consistent with the whole cohort but imprecise (OR 1.42, 95% CI 0.72–2.68; AUC 0.679, 0.153–1.000). For 6-month (17 deaths) and overall mortality (22 deaths) in sAH, IL-6 showed no association (AUC 0.53 and 0.42; MELD-adjusted OR 0.79 [0.45–1.37] and 0.66 [0.36–1.22]). Corticosteroid exposure and Lille response were not available for these patients.

The IL-6 × ALD interaction for in-hospital mortality was unchanged after adjustment for infection and MELD-3.0 (interaction OR 1.93, 1.26–2.98; p = 0.003), after further adjustment for age and sex (1.96, 1.25–3.06; p = 0.003), and after excluding all 30 sAH patients (1.85, 1.19–2.87; p = 0.006). Within ALD, IL-6 discriminated in-hospital death in infected patients (n = 55, 20 deaths; AUC 0.741; MELD-adjusted OR 1.55, 1.00–2.39), in non-infected patients (n = 105, 6 deaths; AUC 0.685; Firth MELD-adjusted OR 1.74, 1.04–3.11) and in ALD without sAH (n = 132, 23 deaths; AUC 0.810; MELD-adjusted OR 1.75, 1.22–2.50). No IL-6 × infection interaction was detected within ALD (p = 0.90) or MASLD (p = 0.38), and no IL-6 × sAH interaction was detected (p = 0.75). In MASLD, IL-6 was not associated with in-hospital death in either infected (n = 82, 5 deaths; AUC 0.551) or non-infected patients (n = 224, 10 deaths; AUC 0.673; MELD-adjusted OR 1.19, 0.73–1.96).

In etiology-stratified mediation analysis (Supplementary Table 5), infection mediated 23.2% of the IL-6–mortality association in ALD (total OR 2.18 → direct OR 1.82, 1.32–2.52; infection → death given IL-6, OR 4.65, 1.56–13.84) but 1.6% in MASLD (total OR 1.27 → direct OR 1.26; infection → death, OR 1.04, 0.32–3.41), and 15.9% in the whole cohort after excluding sAH patients. The incremental gain from adding IL-6 to MELD-3.0 was concentrated in ALD (ΔAUC +0.053, from 0.809 to 0.862; IDI 0.103; likelihood-ratio p < 0.001), was present in ALD with and without infection (ΔAUC +0.042, p = 0.036; +0.104, p = 0.035), and was absent in MASLD (ΔAUC +0.044 but IDI 0.001, p = 0.36), in non-infected patients (p = 0.23) and in patients with the clinical syndrome of ACLF (ΔAUC 0.000, p = 0.31).

**(c) Abstract, Results** after “Infection mediated 17.3% of the interleukin-6–mortality association.” inserted:

In sub-cohort analyses, the etiology-specific effect persisted after adjustment for infection and after exclusion of severe alcohol-associated hepatitis; infection mediated 23% of the interleukin-6–mortality association in alcohol-associated liver disease but 2% in metabolic dysfunction-associated steatotic liver disease, and the incremental value of interleukin-6 over MELD-3.0 was confined to alcohol-associated disease and to infected patients.

## **Comment 6 — RESULTS: explanatory sentences to be moved to the Discussion**

**Comment:** *Explanatory sentence(s): to be moved to Discussion (e.g., 362, 363).*

**Response**

We agree and have modified the sentence structure to reflect true meaning and also prompted by this comment we re-read the Results and removed seven further interpretive clauses; in each case the interpretation is retained where it already exists in the Discussion, and the Results now report findings only. The specific edits are listed below.

**Changes made (deletions and rewordings in the Results)**

- **Section 4.15** (lines 361–363): deleted the sentence “This temporal gradient … as captured by MELD-3.0 over time.”
- **Section 4.7** (lines 248–249): replaced “The attenuation of IL-6's independent effect in the full model was partly attributable to CRP inclusion, which shares substantial variance with IL-6 (ρ = 0.546).” with “Log IL-6 and CRP were moderately correlated (ρ = 0.546).” (The interpretation is retained in the first paragraph of the Discussion.)
- **Section 4.7** (line 263): deleted “, making it a useful “rule-out” threshold” so that the sentence ends “…with a negative predictive value (NPV) of 96.9%.” (Retained in the Discussion, practical-implications paragraph.)
- **Section 4.10** (line 317): deleted “, indicating that in advanced disease, MELD already captures the prognostic information” so that the sentence ends “…overall 87.1% vs. 82.1%, p = 0.609).” (Retained in the Discussion.)
- **Section 4.11** (lines 326–327): deleted “, suggesting that IL-6 captures broader prognostic information beyond what is explained by systemic inflammatory markers alone” so that the sentence ends “…CRP mediated only 9.3% of IL-6's mortality effect.” (Retained in the Discussion, mediation paragraph.)
- **Section 4.14** (lines 344–345): replaced “The concordance of these trimmed analyses with the full-cohort results confirms that findings are not driven by extreme outliers.” with “Estimates were similar to those obtained in the full cohort.”
- **Section 4.15** (lines 370–371): deleted “, a clinically important distinction suggesting etiology-specific utility of IL-6” so that the sentence ends “…P[β > 0] = 77.3%).”
- **Section 4.15** (lines 374–375): deleted “, confirming the complementary roles of disease severity scoring and inflammatory biomarker measurement” so that the sentence ends “…beyond what MELD-3.0 and IL-6 provided.”

## **Comment 7 — DISCUSSION: discuss the “broken” cohort**

**Comment:** *Discuss thus broken cohort (e.g., IL-6 is (is not) of special value in distiguishing patients with infection + sAH, or IL-6 is of special predictive value in patients with sAH without infection at admission...).*

**Response**

A new Discussion paragraph now interprets the sub-cohort analyses. We have been careful to state what the data can and cannot support: the etiology-specific effect is robust to infection and to the exclusion of sAH; infection accounts for a larger share of the IL-6 signal in ALD than in MASLD; and the sAH subgroup (30 patients, three in-hospital deaths) does not allow a conclusion about “special value” of IL-6 in sAH with or without infection, although IL-6 was three-fold higher in sAH with infection and the highest values were seen in the in-hospital deaths. We also report the negative finding that IL-6 was not associated with 6-month or overall mortality in sAH.

**Changes made**

**Section 5 (Discussion)** inserted the following new paragraph immediately after the etiology paragraph ending “…may be less informative about acute prognostic risk.” and before the mediation paragraph (“The mediation analysis provides insight…”):

We examined whether the IL-6 signal is confined to, or modified by, the two precipitants most likely to raise it – bacterial infection and sAH. First, the etiology-specific effect was not an artefact of either precipitant: the IL-6 × ALD interaction was unchanged after adjustment for infection and MELD-3.0 and after excluding all sAH patients, and IL-6 discriminated in-hospital death in ALD with and without infection but not in MASLD in either stratum. Second, infection mediated 23% of the IL-6–mortality association in ALD but 2% in MASLD, where infection itself did not predict in-hospital death once IL-6 was accounted for; in ALD, admission IL-6 thus appears to integrate infection-driven and sterile (alcohol- and gut-derived) inflammation, whereas the weaker, infection-independent signal in MASLD was not clinically useful at our sample size. Third, with 30 sAH patients and three in-hospital deaths, we cannot establish whether IL-6 has special value in sAH with or without infection; the descriptive findings – IL-6 three-fold higher in sAH with infection and highest in the sAH patients who died, are compatible with the whole-cohort results but hypothesis-generating only. IL-6 showed no association with 6-month or overall mortality in sAH, where MELD-3.0 dominated, consistent with the CANONIC study finding that systemic inflammation in acute decompensation varies by precipitant and is most pronounced in alcoholic hepatitis and bacterial infection [31], and with the time-limited admission signal seen in the whole cohort. Overall, IL-6 is most informative in alcohol-associated cirrhosis irrespective of infection, of modest value in infected patients of any etiology (outperforming CRP and procalcitonin but not MELD-3.0 or CLIF-C OF), and of little value in non-infected MASLD or once organ failure is established. Whether IL-6 identifies sAH patients unlikely to respond to corticosteroids, or candidates for early transplantation, could not be addressed here and merits a dedicated study.

## **Comment 8**

**Comment:** *Discuss as I´ve proposed above: “Regular IL -6 use in ... patients is of limited use. Personalized IL-6 value research is warranted in pts with ... as per your results.”*

**Response**

We have adopted the reviewer's framing. The new paragraph (shared with the response to Comment 9, because the two points are inseparable) states explicitly that routine, unselected IL-6 measurement at admission is of limited value, and that personalised IL-6-informed risk stratification warrants further study in patients with alcohol-associated cirrhosis, particularly those with intermediate MELD-3.0 scores and those with documented infection, where the reclassification gain was largest. The Conclusion and the Abstract have been aligned with this statement.

## **Comment 9**

**Comment:** *Alude to the cost-effectiveness of regular IL-6 at admission. Shift the focus on enriched subgroups where IL-6 is worth continued research.*

**Response**

We agree, and the new paragraph below addresses both Comments 8 and 9. We did not collect cost data and have not attempted a formal economic evaluation, which would require modelling of the downstream actions that an IL-6 result might trigger; instead we quantify the “number of patients that would need to be tested” implications of restricting measurement to the enriched subgroups identified in Section 4.16, state the unit cost of the assay in our setting, and call explicitly for prospective studies with embedded economic evaluation. The focus of the practical-implications paragraph, the Limitations, the Conclusion and the Abstract has been shifted accordingly from “IL-6 as a general complement” to “IL-6 as a targeted test in enriched subgroups”.

**Changes made**

**(a) Section 5 (Discussion)**, inserted the following new paragraph immediately after the practical-implications paragraph ending “…may warrant further validation as a simple bedside prognostic tool.” and before “6. Limitations and Future Directions”:

These results could argue against routine, unselected admission IL-6 testing. Cohort-wide gain over MELD-3.0 was small (ΔAUC +0.023) and negligible in non-infected patients (+0.003), MASLD (IDI 0.001) and established ACLF, where organ-failure scores already capture the information. In ALD cirrhosis, however, adding IL-6 to MELD-3.0 raised the AUC from 0.809 to 0.862 (IDI 0.103), and an admission value ≥41.6 pg/mL had 88.5% sensitivity, 31.1% positive and 96.5% negative predictive value for in-hospital death (positive predictive value 9.6% in MASLD). We therefore propose IL-6 as a targeted rather than routine test: of limited value in unselected patients, but warranting personalised risk-stratification research in ALD cirrhosis – particularly at intermediate MELD-3.0 (17–24), where standard scores are least decisive, and with documented infection, where IL-6 outperformed CRP and procalcitonin. The automated immunoassay costs about INR 1200 (USD 13) per test in our setting; formal cost-effectiveness analysis was beyond our scope, but restricting testing to ALD cirrhosis or documented infection (51% of admissions, containing 70% [31/44] of in-hospital deaths) would reduce the tests needed per additional high-risk patient identified, and assay cost must be weighed against the cost and benefit of the actions it may trigger (earlier intensive-care review, intensified infection surveillance, earlier transplant referral). Prospective studies embedding IL-6 in a management algorithm with economic evaluation are needed before any clinical recommendation.

**(b) Section 6 (Limitations)**, after the fifth limitation on cost-effectiveness (“…even when the AUC change appears small.”, inserted:

Because the reclassification benefit was confined to identifiable subgroups, a cost-effectiveness argument for routine measurement cannot be made from these data; the sub-cohort analyses in Section 4.16 were post hoc, involved small numbers of events in several strata, were not corrected for multiplicity, and require prospective confirmation in etiology- and infection-enriched cohorts.

**(c) Section 7 (Conclusion)**, final sentence – replaced “These findings position interleukin-6 as a candidate complement to, not a substitute for, established severity scores for short-term risk assessment, most informative in alcohol-associated and intermediate-severity disease, and warrant confirmation in external, etiology-enriched cohorts before clinical adoption.” with:

These findings position interleukin-6 as a candidate complement to, not a substitute for, established severity scores for short-term risk assessment. Routine measurement in unselected admissions is not supported by these data; targeted use in alcohol-associated cirrhosis – particularly with documented infection or intermediate MELD-3.0 scores – is the setting in which interleukin-6 merits prospective validation with embedded economic evaluation before clinical adoption.

**(d) Abstract, Conclusions** – replaced the final sentence with:

These findings do not support routine interleukin-6 measurement in unselected admissions but identify alcohol-associated cirrhosis, particularly with infection or intermediate MELD-3.0 scores, as the setting in which interleukin-6-informed risk stratification merits prospective validation and economic evaluation.

# **Response to the Additional Editor Comments**

We thank the Editor for pointing us to five recent papers that place our findings in their contemporary context. Each has been read in full, and the additions below were written against the primary sources rather than their abstracts. All five are now cited in the revised manuscript (references 26–30, together with reference 31 which supports the new Discussion material requested by the reviewer).

## **Editor comment 1**

**Comment:** *The Introduction appropriately highlights systemic inflammation as a major determinant of organ failure and short-term mortality in cirrhosis. This section could be strengthened by briefly discussing recent efforts to incorporate inflammation-related variables into conventional prognostic models. In particular, Yu et al. showed that adding parameters such as neutrophil count could improve MELD-based prognostication in ACLF. This study may provide useful contemporary context for the rationale behind evaluating IL-6 as a complementary biomarker (PMID: 39944361).*

**Response**

We agree that this work provides the natural rationale for our study. The cohorts were predominantly HBV-related (86%), and the inflammation variable was a cellular one (neutrophil count). Our study asks the complementary question of whether an upstream cytokine adds information in a non-viral cohort. A sentence to this effect has been added to the Introduction.

**Changes made**

**Section 1 (Introduction), fourth paragraph**, after the sentence ending “…the subgroups in whom IL-6 measurement adds most value to routine prognostic assessment have not been defined.”, inserted:

Recent efforts to improve conventional scores have focused on adding inflammation-related variables. In a meta-analysis of 32 cohorts comprising 13,939 patients with ACLF, followed by validation in 751 prospectively enrolled patients, Yu et al. showed that adding age, neutrophil count and hepatic encephalopathy grade to MELD (the CATCH-LIFE-MELD score) improved short-term prognostication over MELD, MELD-Na and CLIF-C ACLF scores, in derivation and in an external cohort [26]. That work was conducted in predominantly hepatitis B–related ACLF and used a cellular marker of inflammation; whether an upstream cytokine such as IL-6 adds prognostic information in hospitalized patients with non-viral cirrhosis, and in which subgroups, has not been established.

**Reference added** as 16 (Yu et al., eGastroenterology 2024).

## **Editor comment 2**

**Comment:** *When discussing the incremental prognostic value of IL-6 over MELD-3.0 and CLIF-C OF, the authors should place their findings in the context of recent risk-model development in hospitalized patients with cirrhosis. The LIVERAID-ICU score in patients with cirrhosis and infection demonstrated that combining hepatic dysfunction with extrahepatic physiological variables improved the prediction of in-hospital mortality compared with several established liver- and sepsis-specific scores. A comparison with this study would help clarify the potential clinical niche of an IL-6-enhanced model (PMID: 39384247).*

**Response**

We have added this comparison to the paragraph on incremental value. Hoppmann and colleagues derived the LIVERAID-ICU score in 620 patients with cirrhosis treated for infection in a German university ICU (2017–2019); the score combines Child–Pugh class, serum urea and respiratory variables, is calculable at the bedside, and reached an AUC of 0.83 for in-hospital mortality in the validation cohort, exceeding SOFA, MELD, Child–Pugh and CLIF-C ACLF. Our new sub-cohort analysis (Section 4.16) makes the comparison concrete: in our infected patients IL-6 (AUC 0.700) outperformed CRP and procalcitonin but not MELD-3.0 (0.732) or CLIF-C OF (0.749), and the best combined model in the whole cohort was an organ-failure score plus IL-6 (CLIF-C OF + IL-6, AUC 0.802). We therefore frame the clinical niche of an IL-6-enhanced model as an early, upstream inflammatory dimension in patients whose extrahepatic physiological derangement is not yet manifest, particularly in ALD, rather than as a competitor to organ-failure-based scores in critically ill infected patients, where LIVERAID-ICU-type scores already perform well. A direct head-to-head comparison was not possible because the respiratory variables required for the LIVERAID-ICU score were not recorded in our database.

**Changes made**

**Section 5 (Discussion), incremental-value paragraph**, after the sentence ending “…incorporating IL-6 into organ failure–based assessment frameworks yield the most clinically useful prediction.”, inserted:

Our findings should also be placed in the context of recent risk-model development in hospitalized patients with cirrhosis. The LIVERAID-ICU score, derived and validated in 620 patients with cirrhosis treated for infection in an intensive care unit, combined a measure of hepatic dysfunction (Child–Pugh class) with extrahepatic physiological variables (serum urea and respiratory parameters) and achieved an AUC of 0.83 for in-hospital mortality, exceeding SOFA, MELD, Child–Pugh and CLIF-C ACLF scores [19]. Our data point in the same direction, the best-performing combination was an organ-failure-based score plus IL-6 and within infected patients IL-6 outperformed CRP and procalcitonin but not MELD-3.0 or CLIF-C OF. This suggests that the clinical niche of an IL-6-enhanced model is not to replace organ-failure-based or physiology-based scores in critically ill infected patients, where instruments such as LIVERAID-ICU already perform well, but to add an early, upstream inflammatory dimension in patients whose organ-failure burden is still limited, particularly in ALD and at intermediate MELD-3.0 scores, before extrahepatic physiological derangement becomes manifest. A head-to-head comparison with the LIVERAID-ICU score was not possible because the respiratory variables it requires were not captured in our database, and this comparison should be part of any prospective validation.

**Reference added** as 19 (Hoppmann et al., BMJ Open Gastroenterology 2024).

## **Editor comment 3**

**Comment:** *The etiology-specific interaction between IL-6 and alcohol-associated liver disease is potentially the most novel finding of the study, but its mechanistic interpretation could be developed further. It would be beneficial to discuss the contribution of hepatic cell–cell communication and gut–liver and adipose–liver crosstalk to intestinal permeability, microbial translocation, Kupffer-cell activation and amplification of inflammatory signalling in alcohol-associated liver disease (PMID: 39735421).*

**Response**

We have expanded the mechanistic paragraph using the framework set out in the review by Gao and colleagues, which integrates hepatocyte–non-parenchymal cell communication (including extracellular vesicles, cytokines, chemokines and inflammasomes) with the gut–liver axis (dysbiosis, increased intestinal permeability and microbial translocation) and the adipose–liver axis (adipokines and free fatty acid flux). The expanded paragraph now also links this framework to our new mediation finding, a larger infection-mediated share of the IL-6 signal in ALD (23%) than in MASLD (2%), because bacterial translocation and overt infection lie on the same gut-derived pathway.

**Changes made**

**Section 5 (Discussion), etiology paragraph**, replaced the sentence “This is biologically plausible given the distinct inflammatory phenotype of ALD, in which alcohol directly stimulates hepatic macrophages and Kupffer cells to produce IL-6 and other proinflammatory cytokines, promotes gut barrier disruption and bacterial translocation, and induces a more intense neutrophilic inflammatory response.” with:

This is biologically plausible given the distinct inflammatory phenotype of ALD, which recent syntheses of its pathogenesis attribute to coordinated cell-to-cell and organ-to-organ signalling [17]. Ethanol and its metabolite acetaldehyde disrupt intestinal tight junctions and induce dysbiosis, increasing intestinal permeability and the portal delivery of lipopolysaccharide and other microbial products; these engage Toll-like receptor 4 on Kupffer cells, which, primed by chronic alcohol exposure and by extracellular vesicles and damage-associated molecular patterns released from injured hepatocytes, amplify IL-6, tumour necrosis factor-α and CXC-chemokine production and recruit neutrophils into the liver. In parallel, alcohol-induced adipose-tissue lipolysis and inflammation increase the flux of free fatty acids and adipokines to the liver, sustaining hepatocyte stress and macrophage activation [17]. IL-6 sits at the convergence of these intrahepatic, gut–liver and adipose–liver circuits, which may explain why a single admission value carries more prognostic information in ALD than in MASLD, where the inflammatory tone is lower-grade and metabolically driven. This framework is also consistent with our etiology-stratified mediation analysis, in which infection accounted for a larger share of the IL-6–mortality association in ALD (23%) than in MASLD (2%), because bacterial translocation and clinically overt infection lie on the same gut-derived pathway.

**Reference added** as 17 (Gao et al., eGastroenterology 2024).

## **Editor comment 4**

**Comment:** *The discussion of biomarker performance should more clearly acknowledge the broader methodological limitations of prognostic biomarker research in cirrhosis. A recent systematic review covering 63 studies and 49 biomarkers identified substantial heterogeneity in study design, outcome definitions and analytical methodology, and emphasised the need for standardised evaluation and external validation. Although that review focused primarily on compensated cirrhosis, its methodological conclusions are relevant to the present study, particularly regarding the proposed IL-6 thresholds and IL-6/albumin ratio (PMID: 39182920).*

**Response**

We agree and have added this acknowledgement to the Limitations, applying the conclusions of the systematic review by Gananandan, Singh and Mehta (63 studies, 31,438 patients, 49 biomarkers) directly to our own thresholds and to the IL-6/albumin ratio, which were derived post hoc in a single cohort and are optimistic by construction. We also cite the systematic review and meta-analysis of biomarkers in decompensated cirrhosis by Juanola et al. (103 studies, 29 biomarkers), which reached the same methodological conclusions for hospitalized patients while identifying IL-6 as the biomarker with the largest separation between 90-day non-survivors and survivors, which is why we consider IL-6 a reasonable candidate for the standardised, prospective validation that both reviews call for. The wording of the practical-implications paragraph has been softened accordingly (“candidate cut-off” rather than “threshold for risk stratification”).

**Changes made**

**(a) Section 6 (Limitations)**, after the seventh limitation (“Finally, external validation in independent cohorts … clinical practice guidelines.”), inserted:

More broadly, our findings should be read in the light of the methodological limitations that characterise prognostic-biomarker research in cirrhosis. A systematic review evaluating candidate biomarkers found substantial heterogeneity in study design, outcome definitions and analytical methods, frequent reliance on single-centre derivation cohorts and data-driven thresholds, and a lack of external validation, and called for standardised evaluation frameworks [21]. Although that review addressed compensated cirrhosis, its conclusions apply directly to the present study: the IL-6 cut-offs (41.6 and 10 pg/mL) and the IL-6/albumin ratio proposed here were derived post hoc in a single cohort, are optimistic by construction, are assay-dependent, and must be regarded as candidate rather than validated tools until they have been tested prospectively in independent cohorts with prespecified thresholds, harmonised outcome definitions, and assessment of calibration and clinical utility. A systematic review and meta-analysis of biomarkers in decompensated cirrhosis reached similar methodological conclusions while identifying IL-6 as the biomarker with the largest ratio of means between 90-day non-survivors and survivors [22], which supports IL-6 as a reasonable candidate for such validation but does not substitute for it.

**References added** as 29 (Gananandan et al., BMJ Open Gastroenterology 2024) and 30 (Juanola et al., Gut 2024).

## **Editor comment 5**

**Comment:** *The mediation analysis suggests that IL-6 reflects inflammatory processes extending beyond clinically documented infection. The authors could further acknowledge that hepatic and extrahepatic inflammatory responses may be biologically dissociated in ACLF.*

**Response**

We agree. The mediation paragraph now acknowledges that hepatic and systemic (extrahepatic) inflammatory responses may be biologically dissociated in ACLF, that a single serum IL-6 measurement sums hepatic and extrahepatic production and cannot apportion the signal to its source, and that the “direct” effect in our mediation model should be interpreted as inflammation not captured by the clinical diagnosis of infection rather than as hepatic inflammation per se. We cite the CANONIC analysis of systemic inflammation, which showed that the cytokine profile differs according to the precipitating event and that systemic inflammation, rather than hepatic dysfunction alone, tracks ACLF grade and outcome.

**Changes made**

**Section 5 (Discussion), mediation paragraph**, after the sentence ending “…rather than a specific indicator of any single pathological process.”, inserted:

We also acknowledge that hepatic and extrahepatic inflammatory responses may be biologically dissociated in ACLF. Circulating IL-6 is the sum of hepatic (Kupffer-cell and hepatocyte) and extrahepatic (gut-derived, adipose, circulating monocyte and endothelial) production, and the CANONIC study analysis of systemic inflammation showed that the profile of circulating cytokines differs according to the precipitating event, being most pronounced with active alcoholism or alcoholic hepatitis and with bacterial infection, and that the severity of systemic inflammation, rather than of hepatic dysfunction alone, tracks the grade and course of ACLF [18]. A single serum measurement therefore cannot apportion the inflammatory signal to its source, and the direct effect in our mediation model should be interpreted as inflammation that is not captured by the clinical diagnosis of infection, rather than as hepatic inflammation per se; compartment-specific or tissue-level markers would be required to resolve this distinction.

**Reference added** as 18 (Clària et al., Hepatology 2016) — shared with the response to Reviewer Comment 7.

**Supplementary tables added:**

**Supplementary Table 1:** Admission pathway, presenting problems, precipitants, level of care and special populations in the study cohort

| **Settings and patient pathways** | **N (%)** |
| --- | --- |
| Route of admission |  |
| Emergency/unplanned admission | 341 (69.7%) |
| Planned admission via outpatient clinic | 148 (30.3%) |
| Presenting complaint(s) prompting admission (not mutually exclusive) |  |
| Ascites | 148 (30.3%) |
| Suspected/confirmed infection or sepsis | 112 (22.9%) |
| Variceal bleeding | 90 (18.4%) |
| Jaundice | 74 (15.1%) |
| Hepatic encephalopathy | 73 (14.9%) |
| HCC-related | 33 (6.7%) |
| Acute kidney injury | 6 (1.2%) |
| Hyponatraemia | 4 (0.8%) |
| Hypotension | 4 (0.8%) |
| Other/multifactorial | 13 (2.7%) |
| Identifiable acute precipitant recorded | 110 (22.5%) |
| Bacterial infection / septic shock | 43 (8.8%) |
| Severe alcohol-associated hepatitis (sAH) | 30 (6.1%) |
| Complementary and alternative medicine (CAM)-related hepatotoxicity | 26 (5.3%) |
| Drug-induced liver injury (conventional drugs) | 6 (1.2%) |
| Viral hepatitis reactivation/flare (HBV/HCV/acute viral hepatitis) | 7 (1.4%) |
| Other (hypotension, surgery) | 3 (0.6%) |
| Clinical syndrome at admission |  |
| Compensated cirrhosis | 39 (8.0%) |
| Decompensated cirrhosis | 338 (69.1%) |
| Acute-on-chronic liver failure | 112 (22.9%) |
| Level of care and organ support |  |
| Direct admission to medical ICU | 150 (30.7%) |
| Transfer to ICU during hospital stay | 151 (30.9%) |
| Hypotension at admission | 31 (6.3%) |
| Mechanical ventilation at admission / during stay | 15 (3.1%) / 33 (6.7%) |
| Renal replacement therapy at admission / during stay | 9 (1.8%) / 16 (3.3%) |
| Acute variceal bleeding at admission | 91 (18.6%) |
| Documented infection at admission | 145 (29.7%) |
| Special populations |  |
| Hepatocellular carcinoma | 97 (19.8%) |
| Transition to do-not-resuscitate status during admission | 26 (5.3%) |
| Outcomes |  |
| In-hospital mortality | 44 (9.0%) |
| Length of stay among survivors, median (IQR) days | 5 (3–7) |

**Supplementary Table 2: Infections documented at admission: site, organism, IL-6 and in-hospital mortality**

| **Characteristics** | **Value** |
| --- | --- |
| Patients with documented infection at admission | 145 (29.7% of 489) |
| Site of infection (not mutually exclusive; 21 patients had ≥2 sites) |  |
| Bloodstream infection (bacteraemia/fungaemia) | 76 (52.4%) |
| Urinary tract infection | 51 (35.2%) |
| Respiratory tract infection (pneumonia/LRTI, pleural) | 13 (9.0%) |
| Spontaneous bacterial peritonitis | 12 (8.3%) |
| Skin and soft-tissue infection | 12 (8.3%) |
| Bone and joint infection | 3 (2.1%) |
| Infective gastroenteritis | 1 (0.7%) |
| Organism identified (any site) | 99 (68.3%) |
| Klebsiella pneumoniae | 25 |
| Candida spp./other fungi | 18 |
| Escherichia coli | 15 |
| Enterococcus spp. | 5 |
| SARS-CoV-2 | 5 |
| Enterobacter spp. | 4 |
| Staphylococcus spp. | 3 |
| Leptospira spp. | 2 |
| Streptococcus spp. | 2 |
| Pseudomonas aeruginosa | 2 |
| Salmonella spp. | 2 |
| Acinetobacter baumannii | 1 |
| IL-6 (pg/mL), median (IQR): infected vs non-infected | 63.1 (21.6–199.8) vs 17.0 (6.8–46.1); p < 0.001 |
| In-hospital mortality: infected vs non-infected | 25/145 (17.2%) vs 19/344 (5.5%) |
| IL-6 median (IQR) — Bloodstream infection | 91.0 (32.0–323.6) [n=76; in-hospital deaths 17] |
| IL-6 median (IQR) — Urinary tract infection | 46.1 (20.8–90.3) [n=51; in-hospital deaths 3] |
| IL-6 median (IQR) — Respiratory tract infection | 74.6 (45.6–206.5) [n=13; in-hospital deaths 3] |
| IL-6 median (IQR) — Spontaneous bacterial peritonitis | 203.8 (59.6–827.0) [n=12; in-hospital deaths 1] |
| IL-6 median (IQR) — Skin and soft-tissue infection | 39.5 (11.2–146.5) [n=12; in-hospital deaths 2] |

**Supplementary Table 3:** Incremental value of IL-6 over MELD-3.0 and cut-off (41.6 pg/mL) performance by stratum

| **Stratum** | **n (deaths)** | **AUC MELD-3.0 alone** | **AUC MELD-3.0 + IL-6** | **ΔAUC** | **IDI** | **LR-test p** | **IL-6 ≥41.6 pg/mL: sensitivity %** | **Specificity %** | **PPV %** | **NPV %** |
| --- | --- | --- | --- | --- | --- | --- | --- | --- | --- | --- |
| Whole cohort | 489 (44) | 0.762 | 0.785 | +0.023 | 0.0221 | 0.008 | 77.3 | 66.1 | 18.4 | 96.7 |
| Documented infection at admission | 145 (25) | 0.732 | 0.763 | +0.032 | 0.0203 | 0.071 | 92.0 | 42.5 | 25.0 | 96.2 |
| No infection at admission | 344 (19) | 0.746 | 0.749 | +0.003 | 0.0054 | 0.232 | 57.9 | 74.8 | 11.8 | 96.8 |
| ALD (all) | 160 (26) | 0.809 | 0.862 | +0.053 | 0.1031 | <0.001 | 88.5 | 61.9 | 31.1 | 96.5 |
| ALD with infection | 55 (20) | 0.812 | 0.854 | +0.042 | 0.0568 | 0.036 | 95.0 | 40.0 | 47.5 | 93.3 |
| ALD without infection | 105 (6) | 0.604 | 0.707 | +0.104 | 0.0910 | 0.035 | 66.7 | 69.7 | 11.8 | 97.2 |
| MASLD (all) | 306 (15) | 0.623 | 0.667 | +0.044 | 0.0010 | 0.356 | 66.7 | 67.7 | 9.6 | 97.5 |
| MASLD with infection | 82 (5) | 0.757 | 0.758 | +0.001 | 0.0046 | 0.463 | 80.0 | 42.9 | 8.3 | 97.1 |
| MASLD without infection | 224 (10) | 0.765 | 0.762 | -0.003 | 0.0026 | 0.496 | 60.0 | 76.6 | 10.7 | 97.6 |
| ACLF (clinical syndrome) | 112 (16) | 0.788 | 0.788 | +0.000 | 0.0148 | 0.308 | 81.2 | 58.3 | 24.5 | 94.9 |
| sAH (all) | 30 (3) | 0.815 | 0.840 | +0.025 | -0.0024 | 0.535 | 66.7 | 59.3 | 15.4 | 94.1 |

**Supplementary Table 4:** Characteristics and outcomes of sAH vs other ALD vs whole cohort

| **Characteristic** | **sAH (n=30)** | **ALD without sAH (n=132)** | **Whole cohort (N=489)** |
| --- | --- | --- | --- |
| Age, years | 48 (42–54) | 59 (51–66) | 62 (54–68) |
| Male sex | 30 (100.0%) | 130 (98.5%) | 418 (85.5%) |
| Total bilirubin, mg/dL | 10.9 (4.4–19.9) | 5.2 (2.9–10.4) | 3.3 (1.6–6.6) |
| AST, IU/L | 104 (64–150) | 86 (52–137) | 66 (45–115) |
| INR | 2.06 (1.71–2.63) | 1.94 (1.55–2.42) | 1.71 (1.42–2.24) |
| Creatinine, mg/dL | 1.2 (0.9–1.6) | 1.1 (0.8–1.8) | 1.1 (0.8–1.7) |
| MELD-3.0 | 26 (24–32) | 22 (18–28) | 20 (15–26) |
| CTP score | 11 (10–12) | 10 (9–12) | 10 (8–11) |
| CLIF-C OF score | 8 (7–10) | 8 (6–9) | 7 (6–9) |
| Number of organ failures at admission | 2 (2–3) | 2 (1–2) | 2 (1–2) |
| CRP, mg/L | 21.1 (8.8–50.9) | 33.4 (9.6–58.8) | 18.1 (5.8–44.7) |
| Procalcitonin, ng/mL | 0.50 (0.18–1.00) | 0.50 (0.20–1.90) | 0.30 (0.10–0.90) |
| IL-6, pg/mL | 35.1 (18.8–65.5) | 33.5 (12.2–108.3) | 23.8 (9.4–71.8) |
| Documented infection at admission | 10 (33.3%) | 47 (35.6%) | 145 (29.7%) |
| Hepatic encephalopathy at admission | 11 (36.7%) | 39 (29.5%) | 131 (26.8%) |
| Acute kidney injury at admission | 7 (23.3%) | 40 (30.3%) | 103 (21.1%) |
| Direct MICU admission | 9 (30.0%) | 54 (40.9%) | 150 (30.7%) |
| In-hospital mortality | 3 (10.0%) | 23 (17.4%) | 44 (9.0%) |
| 6-month mortality | 17 (56.7%) | 93 (70.5%) | 295 (60.3%) |
| Overall mortality during follow-up | 22 (73.3%) | 98 (74.2%) | 345 (70.6%) |

**Supplementary Table 5:** Baron–Kenny mediation by infection, stratified by etiology

| **Stratum** | **n (deaths)** | **Path c: total effect, OR (95% CI)** | **Path a: IL-6 → infection, OR (95% CI)** | **Path b: infection → death given IL-6, OR (95% CI)** | **Path c′: direct effect, OR (95% CI)** | **Proportion mediated by infection** |
| --- | --- | --- | --- | --- | --- | --- |
| Whole cohort | 489 (44) | 1.61 (1.34–1.93) | 1.69 (1.47–1.95) | 2.08 (1.04–4.19); p = 0.040 | 1.48 (1.22–1.80); p = <0.001 | 17.4% |
| ALD | 160 (26) | 2.18 (1.58–3.00) | 1.83 (1.42–2.35) | 4.65 (1.56–13.84); p = 0.006 | 1.82 (1.32–2.52); p = <0.001 | 23.2% |
| MASLD | 306 (15) | 1.27 (0.94–1.72) | 1.59 (1.33–1.89) | 1.04 (0.32–3.41); p = 0.949 | 1.26 (0.91–1.75); p = 0.156 | 1.6% |
| Whole cohort excluding sAH | 459 (41) | 1.62 (1.34–1.96) | 1.65 (1.43–1.89) | 2.04 (1.00–4.19); p = 0.051 | 1.50 (1.23–1.84); p = <0.001 | 15.9% |
